# The Application of Textile Materials in Interfacial Solar Steam Generation for Water Purification and Desalination

**DOI:** 10.3390/polym16060793

**Published:** 2024-03-13

**Authors:** Haroon A. M. Saeed, Veronica Valerian Kazimoto, Weilin Xu, Hongjun Yang

**Affiliations:** 1 Key Laboratory of Green Processing and Functional New Textile Materials of Ministry of Education, Wuhan Textile University, Wuhan 430200, China; haroonsaeed75@gmail.com (H.A.M.S.); weilin_xu@hotmail.com (W.X.); 2 Textile Engineering Department, Faculty of Industries Engineering &Technology, University of Gezira, Wad Madani P.O. Box 20, Sudan; 3 College of Textile, Wuhan Textile University, Wuhan 430200, China; veronicavalerian123@gmail.com

**Keywords:** interfacial solar steam generation, textile materials, wastewater purification, desalination, woven fabric, electrospun membranes

## Abstract

The global increase in population, the phenomenon of climate change, the issue of water pollution and contamination, and the inadequate management of water resources all exert heightened strain on freshwater reserves. The potential utilization of the interfacial solar steam generation (ISSG) system, which utilizes photothermal conversion to generate heat on material surfaces for wastewater purification and desalination purposes, has been successfully demonstrated. Textile-material-based ISSG devices, including (woven, nonwoven, and knitted) fabrics and electrospinning membranes, exhibit distinct properties such as a rough surface texture, high porosity, significant surface area, exceptional flexibility, and robust mechanical strength. These characteristics, combined with their affordability, accessibility, and economic viability for widespread implementation, make them extremely attractive for applications in SSG. In this review, a comprehensive analysis of the emerging concepts, advancements, and applications of textile materials, such as woven, nonwoven, and knitted fabrics and electrospun membranes, in ISSG for wastewater purification and desalination is presented. We also emphasize significant obstacles and potential prospects in both theoretical investigations and real-world implementations, aiming to contribute to future advancements in the domain of textile-material-based interfacial evaporation in wastewater purification and desalination. Furthermore, the drawbacks and the challenges of ISSG systems are also highlighted.

## 1. Introduction

Although approximately 75% of the surface area of the Earth is covered by seawater [1,2,3], almost 97% of the Earth’s water is saline, meaning that it has a high concentration of dissolved salts [4,5,6,7]. This saline water is primarily found in the oceans and seas, making it mostly unsuitable for direct utilization in most applications, including drinking, irrigation, and industrial processes [8,9]. This leads to a significant proportion, exceeding one-third, of the global populace residing in nations confronting water scarcity [10,11]. And it is expected that this proportion will increase to around two-thirds by the year 2025 [12,13,14]. Based on the United Nations World Water Development Report 2019, it is evident that global human water consumption has escalated to six times its level a century ago. Furthermore, projections indicate that this demand will surge from 20% to 50% by 2050 [15,16]. However, the subject of global freshwater scarcity has been identified as a significant issue [17]. The scarcity of easily accessible drinking water is increasingly posing a significant issue for the advancement and sustainability of functional civilizations [18] due to climate change [3,19], socio-economic issues [20,21], the rapid growth of urbanization [22], industrialization [6,23,24,25], agriculture and animal husbandry [26,27], the expansion of the population [28,29], and escalating levels of water pollution [30,31]. Providing clean drinking water globally is widely recognized as a fundamental requirement for promoting optimal human health. It is reported that the necessary daily water intake for individuals to sustain various physiological processes typically ranges from 2.5 to 7.5 L. Despite the tremendous advancements in technological development, providing a secure and environmentally friendly water supply continues to pose significant challenges, particularly in emerging nations and geographically isolated regions [32]. However, addressing the scarcity of freshwater requires a multifaceted approach. It involves implementing sustainable water management practices, promoting water conservation, investing in infrastructure for water storage and distribution, improving water quality through pollution control measures, and developing innovative technologies for wastewater purification and desalination.

Currently, there are multiple techniques for the desalination of seawater, including membrane distillation [33], reverse osmosis [9,10,19], multistage flash (MSF) technology [31], electrodialysis [34], and solar-driven evaporation [13,17]. However, the use of industrial desalination techniques and wastewater purification is constrained by protracted operations, costly devices, and significant power usage [35]. Solar energy is considered one of the most environmentally friendly options among other sources, such as wind energy, tidal energy, and geothermal energy [6,36]. Recently, interfacial solar steam generation (ISSG) has garnered significant interest and is widely regarded as a promising method for producing pure water in research and industrial settings [37,38]. This is because [36,39] it requires low energy usage and is the cleanest option, as well as a safe one [40]; it has ease of manipulation [16]; it is portable, electricity-independent, and has almost zero carbon emissions [2]; it is inexhaustible [41]; it is an abundant, green, renewable, and sustainable energy resource [9,42,43]; it uses less and free energy, has an elevated water evaporation rate, and has straightforward operational procedures [44]; and it lacks an environmental impact [45].

Recently, many solar vapor devices have been created with the primary purpose of capturing freshwater, and there are many literature reviews available about photothermal conversion materials and the comprehensive architecture of such devices. To the best of our knowledge, there is no review that discusses the role of textile materials in ISSG. Therefore, this review thoroughly analyzes several textile materials, such as woven, nonwoven, and knitted textiles, as well as electrospinning membranes. It examines their specific functions and benefits in wastewater purification and desalination within an ISSG system. Furthermore, this research emphasizes the challenges related to the process of utilizing textile materials in Interfacial Solar Steam Generation (ISSG) systems for wastewater purification. We also deliberate on potential opportunities and suggestions.

### 1.1. Interfacial Solar Steam Generation (ISSG)

Solar energy is a source of clean energy that has high potential due to its abundance, renewable nature, inexhaustibility, and environmentally favorable nature [37,46]; it can be harnessed and transformed into various forms, including light, heat, and electricity [37]. Furthermore, it is widely recognized as a potential energy source in several applications [47], including but not limited to hydrogen production, power generation [48], photovoltaic applications, photocatalysis, desalination [49], and water treatment [50,51]. The ecological problems and high energy usage associated with conventional water purification techniques have spurred the development of alternative technologies [52], such as solar interfacial evaporation. These emerging technologies aim to address the limitations of conventional methods by utilizing renewable energy sources, improving energy efficiency, and minimizing environmental impacts [30]. Generally, the conversion efficiency of ISSG is relativity high (70–100%), while conventional methods of solar evaporation typically exhibit a relatively low photothermal conversion efficiency, ranging from 30% to 45% [15,53], due to inadequate solar absorption and significant heat dissipation resulting from the positioning of the light absorber at the bottom of the water source. Consequently, practical implementation could be improved [46].

**Figure 1:**
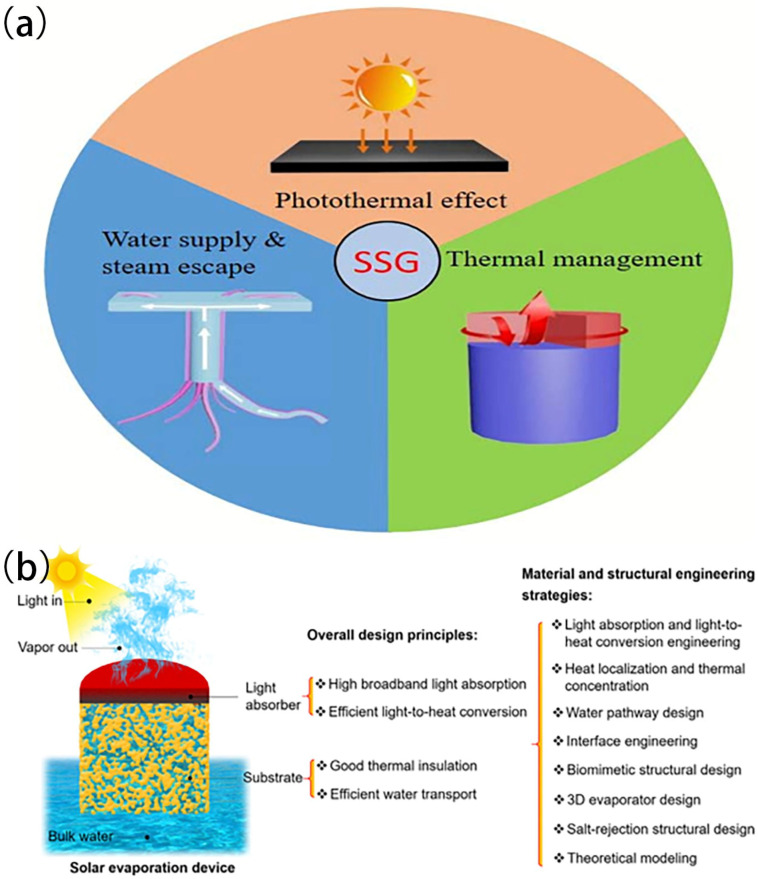
(**a**) Three main factors for ISSG design. Reproduced from ref. [61], copyright 2019, The Royal Society of Chemistry. (**b**) The overall principles and material and designing parameters of SSG system. Reproduced from ref. [46], copyright 2018, Elsevier.

ISSG can effectively harness solar energy to heat the liquid that is located at the interface between the water and air, hence facilitating the formation of steam. Photothermal materials are capable of absorbing and converting solar radiation into heat energy [54]. However, photothermal materials typically have high light absorption properties across a broad spectrum, including visible and near-infrared wavelengths. When exposed to sunlight, the photothermal materials absorb photons and convert them into thermal energy [47]. In order to enhance the effectiveness of interfacial evaporation, inorganic and organic absorbers and photothermal materials, such semiconductor nanomaterials [8,51,55], plasmonic nanoparticles [56], metallic nanoparticles [57], Mxene [58], carbon derivatives [50] such as carbon black [17,29,30,41,53], activated carbon [48], carbon nanotubes [37], carbon fibers [59], and grapheme oxide [22], have been suggested as potential candidates for converting sunlight into heat; in fact, most of these absorbers have been coupled with floating structures of ISSG devices. Among these materials, carbon-based materials exhibit favorable photo stability across a broad spectrum of wavelengths, ranging from 200 to 2500 nm. Additionally, they possess exceptional chemical resistance, rendering them resistant to acidic, alkaline, and saline solutions. Furthermore, their flexible construction capabilities make them desirable for effective solar-driven evaporation applications [60]. The optimization of the solar–vapor conversion performance of an ISSG system requires meeting specific criteria, overarching principles, unique material requirements, and design characteristics, as illustrated in Figure 1a,b. These principles encompass (i) the ability to effectively absorb solar light across a wide range of wavelengths, (ii) the localization of heat at the interface between the air and water, (iii) a sufficient supply of water to sustain continuous evaporation, (iv) the presence of suitable pores that facilitate the avoidance of vapor, and (v) good thermal management properties [21,25].

Photothermal materials are essential for the use of ISSG in water purification. Nevertheless, these materials capture a wide range of solar energy, including visible and near-infrared wavelengths. The light absorber absorbs incident light and converts it into thermal energy. Simultaneously, the substrate absorbs water and conveys it to the evaporative surface via linked water routes that are facilitated by the forces of capillary action. The produced heat causes the water on the evaporative surface to increase in temperature, resulting in a continual evaporation process fueled by a constant water supply. Nevertheless, a portion of the produced heat is unavoidably dissipated to the surrounding environment by being conducted to large amounts of water and air, through radiation to the air, and through convection to large amounts of water. As a result, the efficiency of evaporation is less than 100%. By selecting and designing appropriate photothermal materials, it is possible to boost the efficiency of ISSG devices. This improvement may result in higher rates of steam production, lower energy losses, and expanded practical uses of solar energy for water purification and desalination. Solar interfacial evaporation has the potential to contribute significantly to alleviating global freshwater scarcity, particularly in sunny and water-stressed regions. Ongoing research and development efforts aim to enhance the technology’s efficiency, durability, and cost-effectiveness, making it a viable solution for sustainable freshwater production in the future.

### 1.2. Fundamental Principles for the Design of ISSG Devices

#### 1.2.1. Sunlight Absorption

Solar-driven evaporated water, regarded as a fundamental thermodynamic phenomenon, has been extensively employed in various applications in historical and contemporary contexts, encompassing both natural and industrial settings. The solar-powered evaporation system can be categorized into three categories based on the positioning of the photothermal material within the fluid. The first system under consideration is a conventional bottom-heating-based evaporation method. The second system is a suspended evaporation system. Lastly, the third system is a solar steam generation system [15]. However, solar vapor generation, a process that utilizes solar thermal energy to induce water evaporation without the need for supplementary energy, presents a straightforward approach to addressing the challenges of water purification or desalination [53].

In the conventional bottom heating system, the photothermal material is typically placed at the bottom of the evaporation chamber or on the surface of a heat-conducting substrate. As the fluid is heated, evaporation takes place, and the vapor rises to condense and collect as purified water. This method is commonly used in solar stills or solar desalination systems. Meanwhile, in the suspended evaporation system, the photothermal material is positioned within the fluid, rather than being in direct contact with the bottom of the container. The material is suspended or dispersed within the fluid, allowing for efficient absorption of solar energy. As the material absorbs sunlight, it generates heat, which is transferred to the surrounding fluid, leading to evaporation. On the other hand, the ISSG system is a specialized type of solar-powered evaporation system. It focuses on generating steam rather than directly purifying water. In an ISSG system, the photothermal material is typically positioned at the air–liquid interface. It absorbs solar energy and rapidly converts it into heat, causing localized vaporization at the material’s surface. This vaporization leads to the generation of steam, which can be collected and utilized for various applications like power generation or sterilization.

**Figure 2:**
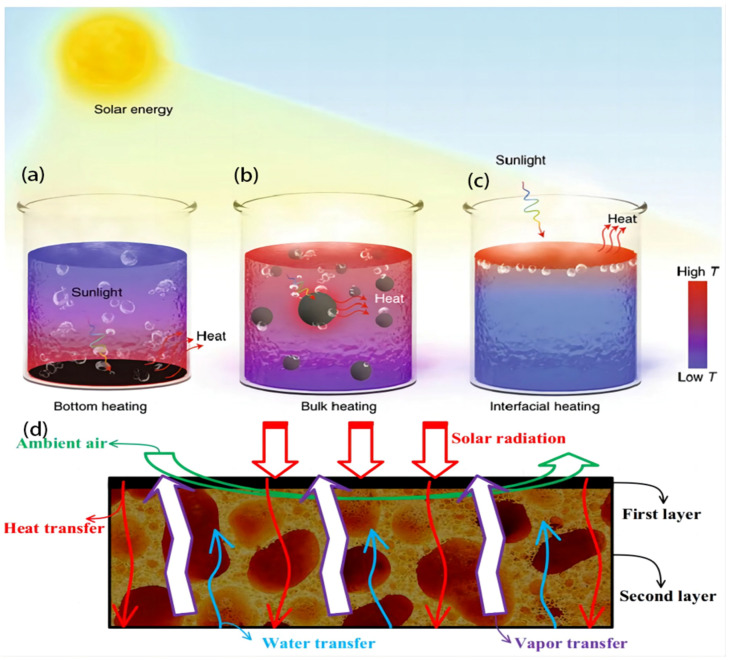
Different ISSG methods of solar heating: (**a**) bottom heating model; (**b**) bulk heating model; (**c**) interfacial heating model. Reproduced from ref. [62], copyright 2023, MDPI. (**d**) The process of the heat and mass transfer in the bottom layer. Reproduced from ref. [63], copyright 2018, Elsevier.

Solar steam systems can be categorized into three distinct heating methods, namely, bottom heating, bulk heating, and interfacial heating, based on the placing of the photothermal substance (Figure 2a–c). The process of bottom heating involves the transformation of solar energy into thermal energy, which is then utilized to immobilize active components at the lower region of a water body. Using this approach causes the application of heat to a significant portion of the aqueous environment. The process of bulk heating involves evenly dispersing plasma nanoparticles, which serve as the photothermal material, across the water body to achieve bulk heating. Interfacial heating refers to placing a photothermal substance at the interface between the air and water, resulting in the concentration of light only on a narrow layer of water on the evaporation surface [62].

ISSG typically involves the use of photothermal materials that absorb sunlight and convert it into heat (Figure 2d). These materials can be incorporated into various configurations such as solar stills, solar evaporators, or solar collectors. When exposed to sunlight, the photothermal materials absorb the solar radiation and heat up, raising the temperature of the surrounding water. As the water reaches its boiling point, it evaporates, leaving behind impurities and contaminants. The vapor is then condensed and collected as clean water, separated from the contaminants [64]. The property of efficient absorption within the sun spectrum is essential when considering a material’s suitability for solar heat localization. The solar spectrum encompasses wavelengths ranging from around 300 nm to 2500 nm. The selected material must possess high absorption efficiency within this specific range of wavelengths, resulting in minimal losses due to reflection and transmission [65]. Obtaining high solar vapor conversion rates is contingent upon two crucial factors: firstly, the maximization of solar energy absorption and heat conversion; and secondly, the reduction in heat loss while improving the distribution of energy for water evaporation [53].

#### 1.2.2. The Principles of ISSG Design

The term photothermal refers to the phenomenon in which the application of light stimulation results in the generation of thermal energy, either partially or entirely. The efficiency of photothermal evaporation is reduced to below 100% due to the inevitable heat loss that occurs because of heat conduction and heat radiation during these procedures. The capacity of a substance to effectively absorb and utilize light is a fundamental factor in the process of photothermal transformation. The two aspects of interest in this context are the extent of absorption across the solar spectrum and the magnitude of absorption at different wavelengths [66]. However, these two requirements can be fulfilled via the selection and design of the photothermal material and understanding of its optical properties, and careful optimization of the absorber design can help extend the absorption across the solar spectrum and improve the magnitude of absorption at different wavelengths, leading to enhanced performance of the ISSG system.

**Figure 3:**
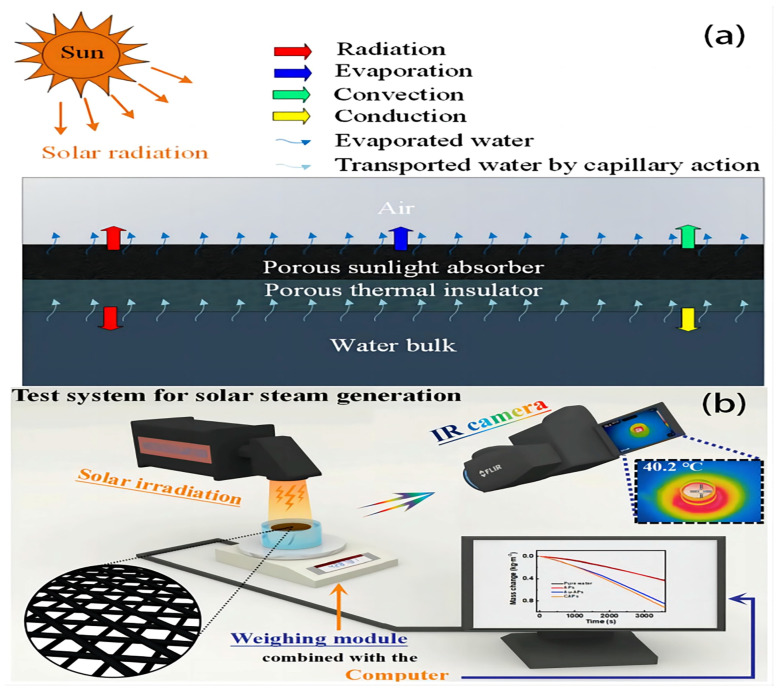
(**a**) The fundamental principles underlying the heat and mass transfer mechanisms within ISSG device. Reproduced from ref. [68], copyright 2018, Elsevier. (**b**) The design for assessing SSG capability. Reproduced from ref. [57], copyright 2018, Frontiers Media S.A.

A set of principles guides the design of an ISSG system to ensure its efficient operation and best performance (Figure 3a). These principles include solar energy absorption, heat localization, water delivery and distribution, heat transfer efficiency, vaporization and evaporation, thermal insulation, system integration and scalability, durability, and longevity, as well as safety and environmental considerations. Nevertheless, these principles serve as a framework for designing ISSG systems, with the aim of maximizing their performance, efficiency, and dependability. By following these principles, ISSG systems may efficiently use solar energy to generate steam and contribute to sustainable energy and water solutions. During a standard measurement process (Figure 3b), the ISSG device is immersed in a container containing either clean water or brine for desalination experiments. Additionally, a solar simulation device is positioned over the apparatus to emit simulated sunlight flux. It is recommended that the size of the light sources be significantly larger than the area of the sample. The level of illumination can be adjusted to any desired power level. However, it is strongly recommended to maintain an illumination level of 1 kW m^2^ (1 sun) due to the dispersed nature of natural sunshine on the planet [67].

The solar-to-vapor conversion efficiency (*η*) was determined by evaluating the evaporation rate by utilizing the following equation [17,52,64,65]:

(1) $$\eta =\frac{\dot{m}h_{LV}}{P_{l}}$$

where *m* is the water evaporation rate (kg m^−2^ h^−1^), *h_LV_* is the total enthalpy of the water–vapor phase conversion, and *ρ*_L_ is the light power density (Wm^−2^). Meanwhile, the evaporation rate can be calculated via the following formula [13]:

(2) $$V=\frac{1}{A\;evap}\;\frac{dm}{dt}=\frac{m}{A\;proj}$$

where the following symbols are used:

ν: evaporation rate (mass of water evaporated per unit time), *m*: mass change of water, *A_evap*: evaporation surface of the system (area over which evaporation occurs), *t*: illumination time (duration of exposure to solar radiation).

#### 1.2.3. Water Supply System in ISSG Devices

During the ISSG operation, water undergoes two primary processes. Firstly, water is absorbed from the reservoir into the photothermal interface. Secondly, water undergoes the process of heating and subsequently transforms into steam, which is then dispersed into the surrounding air. Textiles can efficiently absorb water and transmit steam. To improve the efficiency of evaporation, it is crucial to achieve a balance between the amount of water being provided and the amount of water that has evaporated. Notably, the presence of fibrous materials in the water supply process can reduce the energy that is required for evaporation by decreasing the enthalpy of the evaporated water, hence leading to an increased rate of evaporation [54]. The effectiveness of solar steam generation systems is contingent upon several factors, including their ability to absorb sunlight, move water, facilitate water evaporation, and control temperature swings [69]. The design of water paths is essential for achieving uninterrupted and efficient water movement. Furthermore, the characteristics of the interfaces in solar evaporation systems are also of great relevance. The main attribute of the interface that is of great significance is its wettability. This property is essential in several domains, including water transportation, light assimilation, the buoyancy of devices, and stability. A hydrophilic interface in the outermost layer is desirable due to its capacity to enhance water drainage by capillary pumping. This design technique has been extensively utilized in developing absorber and water transport structures [46].

**Figure 4:**
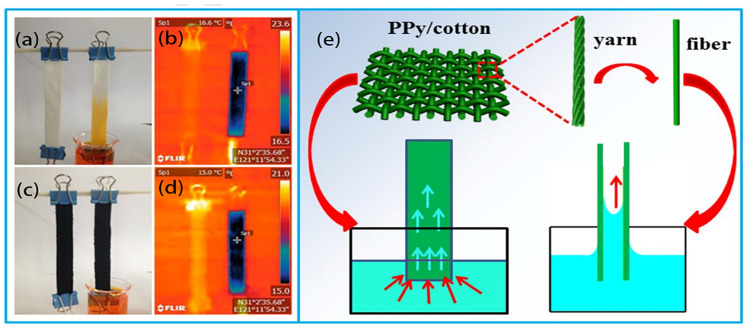
The optical images illustrating the vascular absorption of water by pristine cotton (**a**) and PPy/cotton. (**c**) IR photos demonstrate the temperature variations in pristine cotton (**b**) and PPy/cotton, (**d**) both before and after capillary absorption. (**e**) The process of capillary water absorption in PPy/cotton fabric. Reproduced from ref. [72], copyright 2018, Elsevier.

In textile materials, the spacing between fibers within the material is established through the arrangement of individual fibers that twist and intertwine with neighboring fibers, creating pathways for the transportation of water and steam. However, the dimensions of the porosity and the level of thickness of the fabric are critical variables in ascertaining the water supply. Pores with higher dimensions can facilitate enhanced water and steam transportation, while pores with smaller dimensions can augment the capillary forces [54]. Using hydrophilic fabrics in ISSG systems offers several advantages which highly enhance the overall performance of the ISSG system. However, hydrophilic fabrics have a high affinity for water, allowing them to efficiently absorb and retain water. Improved water absorption ensures a steady supply of water for evaporation and steam generation, leading to higher overall system performance. Moreover, the increased surface area of the textile fabrics provides more contact points for water molecules to interact with solar energy, facilitating faster evaporation rates. However, as water is absorbed by the fabric, it forms a thin film or layer on the surface. This water film acts as a thermal barrier, reducing heat conduction and minimizing heat loss to the surroundings. By reducing heat loss, hydrophilic fabrics contribute to a higher energy conversion efficiency and overall system effectiveness. Textile products such as cotton fabric have garnered significant attention recently due to their inherent hydrophobicity, remarkable gas permeation, exceptional mechanical characteristics, flexibility, and potential for scalability [70], with a strong capillary effect [58]. However, the presence of a hydrophilic surface is of the utmost importance in the process of high-efficiency solar-driven water vapor generation [71]. This is because evaporation occurs primarily at the interface between the air and liquid. This was reported by Hao D. et al. [72], who conducted a study on the effective generation of solar water vapor, facilitated by the utilization of a water-absorbing polypyrrole-coated cotton fabric that exhibited improved thermal localization (Figure 4). The selection of a cotton fabric in this context was based on its hydrophilic properties, inherent porosity structure, widespread availability, favorable mechanical stability, and cost-effectiveness. The results confirmed that a PPy/cotton foam structure demonstrates a solar thermal conversion efficiency of 82.4% when exposed to 1 kW/m^2^ illumination, with an elevated evaporation rate of 1.2 kg m^−2^ h^−1^. The solar vapor generator demonstrated consistent reusability in a series of 30 cycle trials conducted under solar irradiation of 1 kWm^−2^. The findings suggest that the implemented interfacial water evaporation system could play a beneficial role in alleviating the problem of freshwater scarcity.

The utilization of hydrophilic textile materials in ISSG systems augment water absorption, heat transfer efficiency, and performance. Their characteristics enhance energy conversion, minimize heat dissipation, decrease dehydration, and mitigate scaling problems. Through using hydrophilic textiles, ISSG systems may attain enhanced efficacy, heightened rates of water evaporation, and enhanced sustainability in solar steam generation applications.

## 2. Role of Textile Materials in ISSG Systems

Textile materials find extensive application in various sectors, including fashion, sportswear, home textiles, medical textiles, automotive textiles, and many more, Dueto their combination of thinness, lightweight nature, flexibility, comfort, and high degree of flexibility. Additionally, they exhibit a porous structure that can be easily scaled up for industrial production [73]. Furthermore, they demonstrate excellent durability, and the capacity to be programmed to achieve desired pore structures makes them highly suitable for a wide range of products and purposes [74]. Recent advancements in the field of water transmission through textile materials of varying dimensions have provided novel approaches for the development of floating structures aimed at water evaporation. These include techniques such as utilizing the hanging of (woven, nonwoven, knitted) fabrics and composite [41] and electrospun membranes [3]. However, this technique involves suspending or hanging fabric materials over a water surface. The fabric acts as a medium for water transmission and evaporation. As water molecules evaporate from the surface, they pass through the fabric, allowing for continuous evaporation.

The structure of the fabric provides a large surface area for evaporation, enhancing the efficiency of the process. Additionally, the following unique features of the textile materials render them highly promising for solar interfacial evaporation: (i) The fabrics have a textured and rough surface, which enhances their light-absorption abilities. The roughness increases the surface area that is available for solar radiation absorption, allowing for efficient conversion of solar energy into heat. (ii) Textile materials typically have high porosity and a significant surface area. These characteristics provide an extensive evaporation area, allowing for a larger interface between the liquid and the surrounding air. Additionally, the porosity of textiles creates multiple pathways for the steam to escape, reducing the chances of re-condensation. (iii) Textile materials are known for their exceptional flexibility and robust mechanical strength. They can be easily manipulated and shaped into desired configurations, allowing for the creation of portable and scalable devices. (iv) Fabrics are typically cost-effective and readily available materials. The affordability of textiles ensures that the technology can be more accessible and economically viable for widespread implementation, especially in areas with limited resources [54,75]. Moreover, textile materials possess a wide range of raw ingredients, can be produced by a diverse array of adaptable manufacturing methods, and exhibit a high degree of flexibility [54].

The absorbers that are employed in a solar interfacial evaporator are fabricated utilizing a diverse range of substances, including foams, aerogels [11,32,52,69], hydrogels [76], films, membranes [2,77], biomaterials, and textiles [58]. Within this assortment of materials, there is a range of textile materials, including woven fabrics [45,75], knitted fabrics, nonwoven fabrics [71], composite materials [78], and others. Textile materials possess the characteristic of having an adjustable texture and including micronized pores [71], enabling the effortless cleansing of soiled fabric. Moreover, these fabrics exhibit commendable endurance, facilitating the effective elimination of impurities [79]. The characteristics mentioned above have piqued many researchers’ curiosity in advancing adaptable and launderable photothermal textiles that possess micronized pathways and incorporate the encapsulation of photothermal nanoparticles.

### 2.1. Woven Fabrics

**Figure 5:**
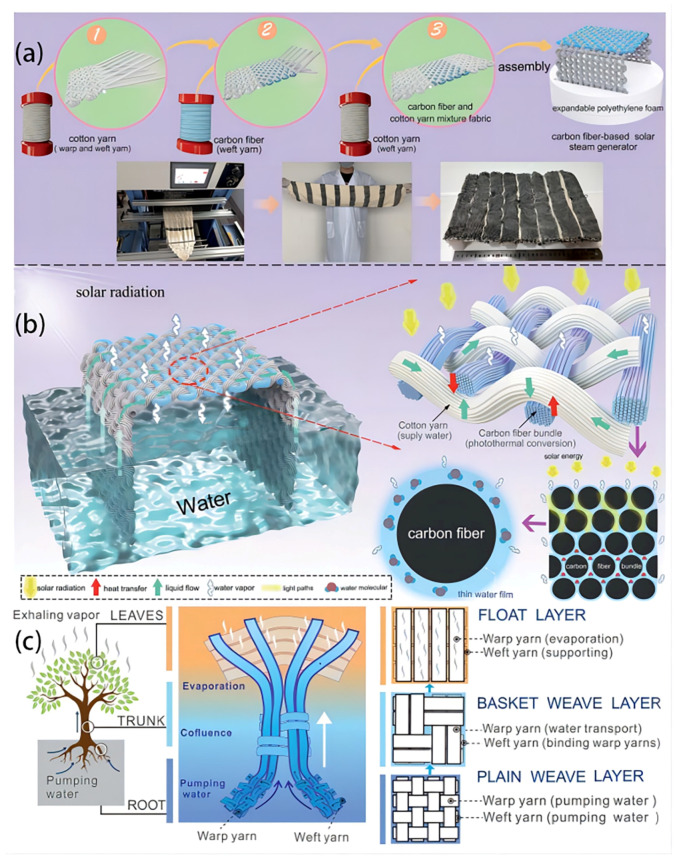
(**a**) The manufacturing of the CCMF-based ISSG including the design of CCMFs by a textile weaving technique. (**b**) The operation concepts of CCMF-based ISSG. Reproduced from ref. [75], copyright 2020, The Royal Society of Chemistry. (**c**) Schematic illustration of the tree shape and the representative biomimic design of the TBFF. Reproduced from ref. [45], copyright 2021, The Royal Society of Chemistry.

Woven technology is usually applied to produce woven fabrics through the systematic interlacing of warp and weft threads, resulting in fabrics that exhibit favorable attributes such as a high tensile strength, resilience to wear and tear, dimensional stability, and a dense structure [54]. Textile products possess a modifiable structure and contain micronized pores, enabling the effortless cleansing of filthy garments. Additionally, these fabrics exhibit commendable longevity, facilitating the effective elimination of impurities [79]. However, cotton has become known as the primary natural fiber being utilized in the textile industry due to its inherent characteristics such as natural softness, significant hygroscopicity, exceptional wear comfort, and skin-friendly properties [80]. By employing a practical textile weaving method, Zhang Q. et al. [75] presented an ISSG device composed of a blend of carbon fiber and cotton yarn (Figure 5a,b). This device has remarkable capabilities in generating solar steam, owing to the carbon fiber’s reliable and efficient light-absorbing properties, as well as the unique structure of the fabric. The manipulation of the woven fabric’s design can control the levels of light absorption and amount of water within the cloth. Under 1 unit of sun light, the modified fabric demonstrates a notable efficiency rate of 1.87 kg m^−2^ h^−1^ and evaporation efficiency of 83.7%. An all-encompassing and expansive apparatus, equipped with anti-salt clogging capabilities, may be readily achieved through the manipulation of the warp thread count and weft thread width. The constructed fabric exhibits considerable promise in the realm of obtaining cost-effective purification of seawater and other forms of wastewater. Consequently, it offers a novel avenue for the advancement of solar interfacial evaporators that are characterized by their high efficiency, scalability, and stability.

The use of three-dimensional (3D) design technology has the potential to revolutionize the manufacturing process of ISSG. Using a conventional loom, Li Y. et al. [45] created a 3D hierarchical tree-shaped biomimetic flax fabric (TBFF) (Figure 5c). The design comprised a float layer, a basket weave layer, and a plain weave layer. The fabric exhibited vertical water transport properties along the uninterrupted warp strands. Subsequently, the TBFF that was obtained underwent a one-step manufacturing process to produce polydopamine–polypyrrole hybrid (PDA-PPy) nanofibers with a larger surface area and enhanced hydrophobicity. However, the fabricated design demonstrated an ordered structure of micro-capillary porosity within the fibers and macro-interlaced pore spaces across the warp and weft threads. This unique design enabled the system to display a wide spectrum of light absorption, efficient water supply, a wide evaporation area, and easy steam escape. Hence, the interconnected water transport pathways established by TBFF-PDA-PPy demonstrated a notable evaporation rate of 1.37 kg m^−2^ h^−1^, with a solar energy transformation performance of 87.4% when subjected to 1 sun irradiation. Due to its straightforward fabrication method, cost-effectiveness, and potential for scalability in manufacturing, the device has potential for facilitating widespread implementation in water filtration and saltwater desalination.

Additionally, a novel approach to fabric draping that involves the separation of the evaporation interface from the bulk water was designed by Gao C. et al. [17]. The drape fabric was coated by a mixture of carbon black (CB) and crosslinked sodium alginate (SA) onto ramie fabric (CSRF). The CSRF exhibited an evaporation rate of 1.81 kg m^−2^ h^−1^ and an efficiency of 96.6% when subjected to 1 sun irradiation. Furthermore, manipulating the yarn fineness in the fabric created an adjustable water supply system, thereby optimizing the energy distribution. This study presents a novel approach to designing and optimizing solar evaporation systems, showcasing significant promise for practical implementation.

**Figure 6:**
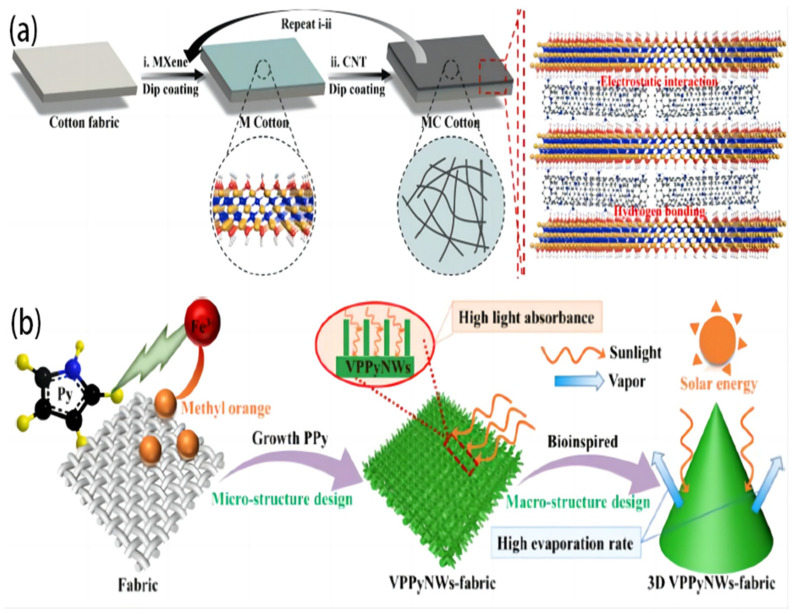
(**a**) Diagram of L-b-L fabrication of MXene nanosheets and CNTs on cotton fabric. Reproduced from ref. [58], copyright 2020, Elsevier. (**b**) Fabrication of 3D VPPyNWs fabric. Reproduced from ref. [81], copyright 2021, American Chemical Society.

A composite fabric combines two or more fibers or materials to create a single fabric with improved qualities. The blended fabric’s combination of materials enables the effective utilization of each component’s capabilities, leading to a fabric that exhibits enhanced performance qualities. Wang Y. et al. [58] constructed a novel composite material of MXene, carbon nanotubes, and cotton fabric using a layer-by-layer assembly technique (Figure 6a). This fabrication aimed to develop a material that is capable of ISSG for purifying textile effluent. The device significantly enhanced the optical absorption, light-to-thermal conversion, and water transport capabilities due to its significant interfacial contacts. Under 1 solar illumination, the evaporation rate for pure water was 1.35 kg m^−2^ h^−1^, while for textile wastewater, it exceeded 1.16 kg m^−2^ h^−1^, with an evaporation efficiency of 88.2%. These values are higher than those observed in prior research on fabric-based composites. Reducing the number of organic–inorganic pollutants in condensed fresh water is a crucial aspect that is often overlooked but holds significant importance in practical applications. In addition, the reusability test and outdoor experiment were conducted to showcase the practical application of the photothermal features. Furthermore, the findings of this study indicate that the composite material possesses superior performance and durability, as well as a straightforward fabrication procedure.

Three-dimensional design allows for the creation of complex geometries and intricate structures that can optimize solar energy absorption and heat transfer within an ISSG system. By leveraging 3D printing techniques, innovative designs can be developed to enhance the overall performance and efficiency of the system. Moreover, the ability to design and manufacture components with precise dimensions, shapes, and features allows for optimized integration with other system elements, maximized energy absorption, and improved overall performance. By incorporating specific patterns, textures, or geometries, 3D-designed components can concentrate solar energy in targeted areas, promoting localized heating and enhancing the efficiency of water evaporation and steam generation. Three-dimensionally printed components can reduce the weight, size, and overall system footprint. This is particularly advantageous for portable or space-constrained applications of ISSG. These characteristics render it a promising option for wastewater purification using solar evaporation. A three-dimensional (3D) ISSG device in the shape of a cone was developed by He M. et al. [81]. The device consists of a cotton fabric coated with vertical polypyrrole nanowires (VPPyNWs) (Figure 6b). However, the polypyrrole (PPy) microstructure enhances solar energy absorption by facilitating simultaneous reflections between the vertically aligned PPy nanowires (VPPyNWs). The overall architecture of the 3D evaporator is characterized by an increased surface area, which promotes efficient energy harvesting, a comprehensive pathway for the water supply, and an open structure that facilitates vapor diffusion. The 3D VPPyNW-fabric-based SSG, developed to demonstrate its feasibility, exhibits a rapid rate of water evaporation of 2.32 kg m^−2^ h^−1^. Additionally, it showcases a high solar absorption performance of 97% and a solar-to-vapor conversion efficiency of 98.56% when subjected to an energy density of 1 kW m^−2^. Furthermore, the SSG can be effectively utilized in diverse water conditions, such as seawater, dye wastewater, and acidic and alkaline wastewater. The effective use of solar energy can be significantly enhanced by implementing a high-performance evaporator incorporating a 3D macro- and structural design. This innovative approach presents a promising opportunity for improved solar energy utilization.

A cost-effective and reusable fabric-based SSG device was successfully fabricated by Kou H. et al. [34] by applying carbon nanotube (CNT)-based ink to conventional cotton fabrics. The cotton fabrics that were colored with a CNT demonstrated significant optical absorption within the wavelength range of 250–2500 nm, resulting in a total solar absorption efficiency of 95.7%. However, when utilizing a polystyrene (PS) foam as the thermal insulator, the cotton–carbon nanotube fabrics with insulating properties demonstrated a notable evaporation rate of saltwater of 1.59 kg m^−2^ h^−1^. Additionally, the apparatus possesses the capability to undergo cleaning and recycling processes through the elimination of the salts that are generated. Following evaporation, the liquid is effectively removed using a straightforward hand-washing procedure. This study demonstrated the feasibility of achieving cost-effective, highly efficient, and large-scale seawater desalination using solar irradiation.

**Figure 7:**
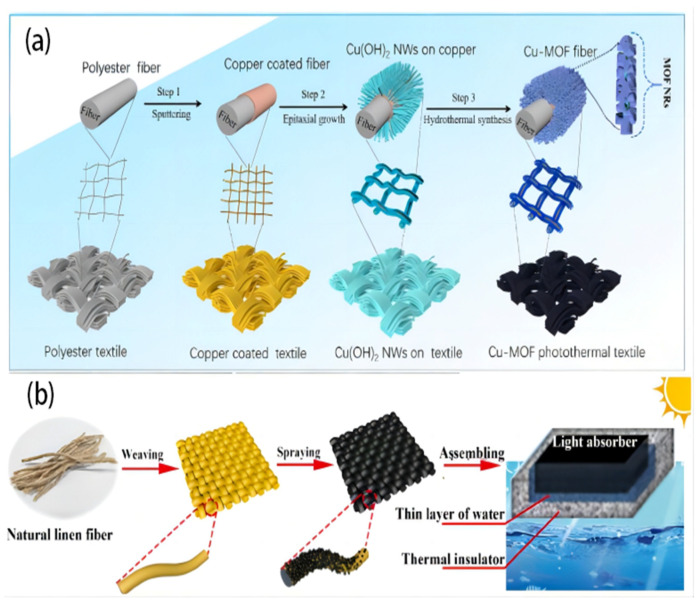
(**a**) Schematic illustration of the synthetic process for CMPT. Reproduced from ref. [82], copyright 2021, Elsevier. (**b**) The natural linen fiber, the original linen fabric (LF), the LF covered with candle soot (LFS), and the integrated LFSTM composed of LFS and a thermal management section (polystyrene). SEM images of original linen fiber. Reproduced from ref. [31], copyright 2020, American Chemical Society.

Hydrophilic fabrics are designed to quickly wick away moisture from the skin, spreading it across a larger surface area to enhance evaporation and promote a dry and comfortable feeling. A highly hydrophilic, linear, and durable copper-based metal–organic framework (Cu-MOF) was designed by Wang J. et al. [82]. An absorber coating was securely attached to the surface of a commercially available textile material through the utilization of a sputtered copper film as a bonding layer (Figure 7a). However, the Cu-MOF photothermal fabric based demonstrated effective prevention of salt formation on its upper surface, even when exposed to a salinity of 9.5 wt% for over 12 h during evaporation. This can be attributed to the CMPT’s exceptional capillary effect and strong water-pumping performance. The CMPT, characterized by its exceptional light absorbance (95.9%) resulting from the distinctive hierarchical structure of Cu-MOF that enhances light trapping and its excellent air permeability, demonstrated a significant evaporation rate of 1.52 kg m^−2^ h^−1^ under 1 solar irradiation. The CMPT has a remarkable structural design, resulting in a high degree of adaptability, exceptional mechanical strength, and superb chemical stability, even in demanding conditions. The study introduces an easy and uncomplicated method for surface immobilization of MOF coatings on textile fibers. This method has potential practical applications in the development of portable solar steam generators. In another study conducted by Song L. et al. [31], an innovative method was used for developing an effective ISSG using a linen fabric with candle soot (Figure 7b). The system combines broadband light absorption, super-hydrophilicity, and an exceptional transition layer of water between a solar evaporator and an insulating material. This design aims to optimize the temperature control and water supply synergistically. By integrating improved energy management techniques and ensuring an ample water supply, the developed ISSG demonstrates a commendable evaporation rate of 1.44 kg m^−2^ h^−1^ and a high efficiency of conversion. The solar irradiation of 1 sun irradiation resulted in a solar-to-thermal conversion efficiency above 90% in salt water with a concentration of 20 wt%. The study has the potential to introduce a novel and straightforward approach to the water purification field.

**Figure 8:**
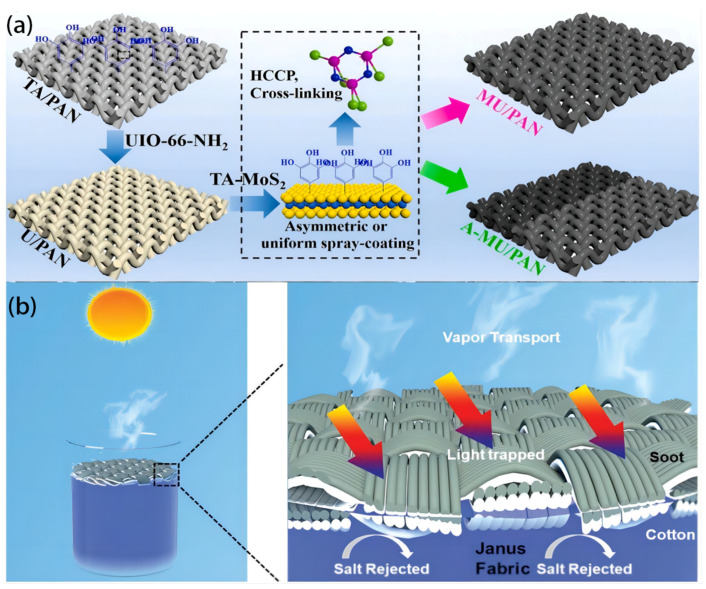
(**a**) Fabrication process of MU/PAN fabric. Reproduced from ref. [83], copyright 2021, Elsevier. (**b**) Design of the soot-deposited Janus fabric floating on the water surface. Reproduced from ref. [84], copyright 2019, WILEY.

In a study by Peng H. et al. [83], a new device for fluidic evaporation was developed, which exhibited asymmetry and enabled edge-preferential crystallization. The device facilitated gravity-assisted salt collection and drenching-induced electrokinetic power generation; it was fabricated by spreading TA-MoS_2_ nanosheets unevenly on a UIO-66-NH_2_-modified PAN fabric, as shown in Figure 8a. The system utilized self-manipulated saline water transport to derive benefits and effectively isolate the crystallized salts from the evaporation surface, facilitating uninterrupted vapor generation and salt extraction for 60 h in sun desalination processes involving saline solutions with a concentration of 7.5 wt%. This wet-fabric evaporator achieved a sustained voltage generation of 0.568 V, solely through saline-drenching, by utilizing the established gradient electric double layers in asymmetric nanochannels. In addition, the evaporator exhibited a high capacity for effectively removing organic pollutants, with minimal impact on both the water evaporation and power generation rate, even after 60 h of continuous operation and 30 cleaning instances, with an evaporation rate of 1.36 kg m^−2^ h^−1^ and a high efficiency of conversion of about 89.2%. This study showcases a scalable multifunctional asymmetric solar evaporator that enables continuous seawater desalination, facilitating salt harvesting and electricity production. Furthermore, it contributes to the progress of adaptable and environmentally friendly applications in practical seawater desalination, specifically for recovering resources, power generation, and storage.

The fabrication of a self-floating Janus cotton fabric coated with a polyester woven fabric, which can reject salt, was successfully designed by Gao S. et al. [84] for an ISSG system (Figure 8b). The device, with controlled soot deposition, exhibits solar absorption properties and the necessary super-hydrophobicity to enable flotation on water, and the Janus evaporator demonstrates a sustained evaporation rate of 1.37 kW m^−2^ h^−1^ and achieves an efficiency of 86.3% when subjected to 1 sun irradiation. Furthermore, it exhibits satisfactory performance under conditions of minimal intensity and tilted radiation. The foldable Janus absorber, which boasts a cost of less than USD 1 per square meter, exhibits great potential as a portable solar vapor generator.

**Figure 9:**
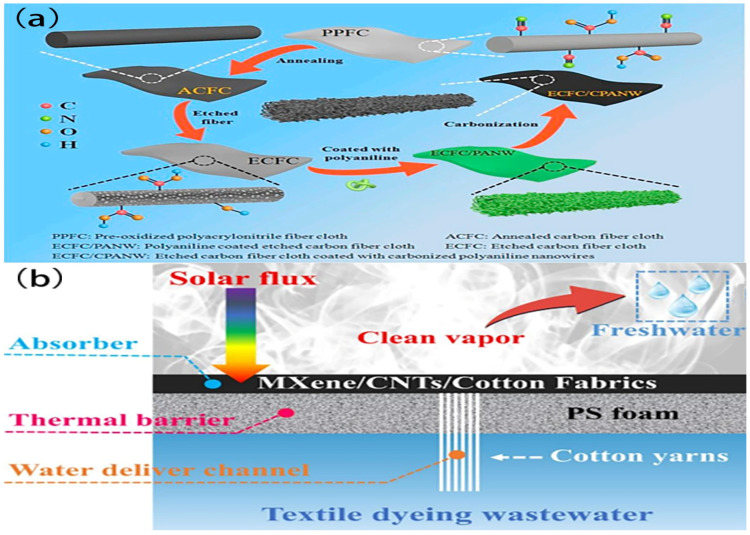
(**a**) The preparation of ECFC/CPANW SSG device. Reproduced from ref. [86], copyright 2019, Elsevier. (**b**) MXene/CNTs/cotton fabric ISSG device. Reproduced from ref. [58], copyright 2020, Elsevier.

Drawing inspiration from the water absorption of the Janus device that was observed in lotus leaves, Qin Z. et al. [85] developed a bio-inspired combination consisting of a cotton fabric supplemented with carbonized carrot powder (CC powder) and coated with Nafion on one side (cotton cloth–NCC). This system was designed to achieve enhanced efficiency in SSG. The utilization of CC powder in the cotton fabric–NCC material serves to enhance light absorption, hence achieving a heightened level of light absorption. Simultaneously, the hydrophilic nature of the cotton cloth facilitates effective water transport. In the interim, applying a Nafion coating creates a Janus architecture with hydrophobic and hydrophilic properties. This structure serves the dual purpose of regulating the water supply and inhibiting salt deposition, even when exposed to high-concentration salt solutions. The cotton cloth–NCC material has undergone further processing to form a waved structure, resulting in w-cotton cloth–NCC. This modification aims to enhance the surface area that is available for water evaporation and obtain a high light absorption level, specifically reaching 95%. When exposed to solar irradiation, the utilization of the w-cotton cloth–NCC material results in a water steam generation rate of 1.88 kg m^−2^ h^−1^ and a saltwater evaporation rate of 1.52 kg m^−2^ h^−1^. In addition, the w-cotton cloth–NCC material has a notable efficacy in purifying sewage. It effectively eliminates Escherichia coli, achieving 100% removal, while demonstrating a high removal rate of 98.3% for Rhodamine B. The methodology proposes a straightforward technique for fabricating a hydrophobic–hydrophilic Janus solar steam evaporator that is characterized by excellent efficiency, inexpensiveness, environmentally friendly operation, and long-term stability. This innovative design exhibits significant potential for use in various fields, including environmental purification and photothermal conversion. Additionally, a novel ISSG device was developed by Wang K. et al. [86] by using a thermally treated pre-oxidized polyacrylonitrile fiber fabric combined with carbonized polyaniline nanowires (ECFC/CPANW), as shown in Figure 9a. The system exhibited several advantageous characteristics, including low thermal conductivity, efficient absorption of solar radiation across a wide range of wavelengths, favorable hydrophilic properties, and excellent water transport capabilities. The results showed a remarkable vaporization efficiency of up to 93.7% with an evaporation rate of 1.43 kg m^−2^ h^−1^ when subjected to 1 sun irradiation. Hence, using cost-effective, stable, and environmentally friendly photothermal conversion materials holds significant potential for real-world use in water purification processes. A novel composite membrane of MXene, carbon nanotubes, and cotton fabric was fabricated by Wang Y. et al. [58] by using a layer-by-layer construction technique for an ISSG device (Figure 9b). Due to its robust surface contacts, the hybrid fabric significantly enhanced optical absorption, light-to-thermal conversion, and water transport capabilities. The evaporation rate for water was found to be 1.35 kg m^−2^ h^−1^, while for textile wastewater, it exceeded 1.16 kg m^−2^ h^−1^ under 1 solar illumination. In addition, the reusability test and outdoor experiment were conducted to showcase the photothermal characteristics of the material when implemented in real-world scenarios. The results indicate that the combined material possesses favorable characteristics such as excellent performance and durability, as well as a straightforward fabrication technique. Thus, it holds promise as a viable option for wastewater purification by solar evaporation methods.

Peng H. et al. [69] presented a novel approach to purify seawater and produce power by developing a one-way asymmetrical nanofluidic photothermal evaporator. The design of this evaporator is inspired by the water and solute transportation in plants, specifically through the transpiration process. The fabrication process involves the in-proportion deposition of photothermal MXene nanosheets on a hydrophilic cotton fabric. However, the cotton fabric pump facilitates the linear transportation of a saline solution, allowing for self-operating salt rejection and stable steam generation in the evaporator. Additionally, forming an asymmetric double electrode layer within MXene nanochannels under the drenching state enables continuous electric power generation. The solar-induced evaporation rate and voltage generation achieved a value of 1.38 kg m^−2^ h^−1^, with an efficiency of 83.1% and 363 mV, respectively, when exposed to 1 sun irradiation. Significantly, the nanofluidic system exhibits minimal degradation in function following 30 h of usage and undergoing 15 washing cycles. This observation underscores its exceptional stability and capacity for repeated use. The straightforward design of the asymmetric nanofluidic photothermal system presents potential opportunities to expand sustainable freshwater and electric power generation.

**Figure 10:**
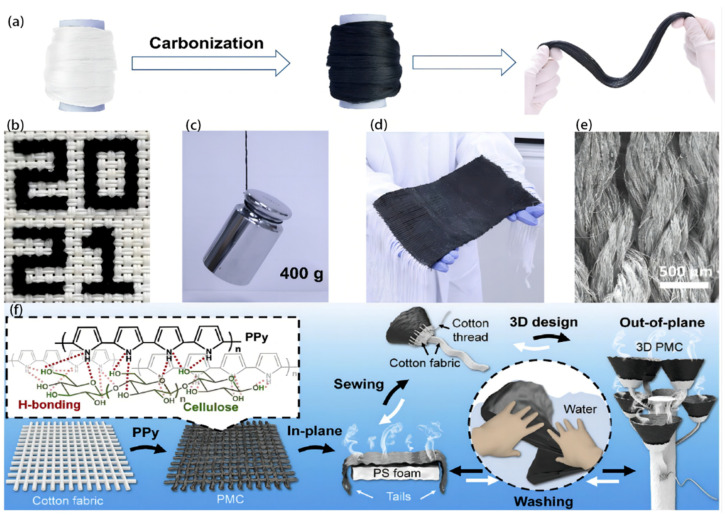
(**a**) Photos of *Bombyx mori* (*B. mori*) silks before and after the carbonization; (**b**) “2021” is knitted by carbonized *B. mori* silks (CBSs); (**c**) a load of 400 g is lifted by CBSs (0.1 g); (**d**) a fabric is made of CBSs and cotton yarns; (**e**) the SEM image of a composite fabric. Reproduced from ref. [87], copyright 2021, Fronitiers. (**f**) Fabrication of functionalized cotton fabric as 2D/3D SSG. Reproduced from ref. [88], copyright 2019, Elsevier.

Carbon fibers derived from two types of animal silks, namely, *Bombyx mori* (*B. mori*) silk and *Antheraea pernyi* (*A. pernyi*) silk, were fabricated by Qi P. et al. [87] by using a single-step heating process on a massive scale without the need for any additives or activation procedures. The carbon fibers and yarns possess both electrical conductivity and mechanical strength. To enhance the utilization of carbonized silks, they proceeded to interlace them with cotton yarns, resulting in the production of composite fabrics with diverse patterns (Figure 10a–e). The results verified the benefits associated with using hybrid fabrics, including enhanced light absorption, increased surface area, and hierarchical liquid transfer routes. These advantageous characteristics make these fabrics suitable for applications such as SSG for desalination and sewage treatment. Furthermore, it was observed that these conductive carbon fibers can be organized into fluidic nano-generators, enabling the generation of electrical energy by the movement of water.

Conventional photothermal materials primarily address concerns related to energy efficiency and multipurpose use. However, the absence of adjustable, affordable, adaptable, and washable materials significantly hampers their transition from laboratory settings to industrial applications. The achievement of enhanced energy efficiency is of great importance because of the limited utilization of spatial volume in traditional 2D evaporators. Consequently, there is a strong need for the advancement of customizable and programmable 3D designs. Xiao P. et al. [88] fabricated a functional photothermal fabric that draws inspiration from conventional sewing techniques. The substance exhibits exceptional scalability, washability, and affordability. It enables the creation of tunable and programmable 2D/3D structures, facilitating efficient water extraction from liquid and solid mediums in both in-plane and out-of-plane directions. An in situ adjustable oxy polymerization approach was used to fabricate cotton fabric modified with polypyrrole (PPy) due to the favorable photothermal properties, stability, and effective contacts exhibited by PPy with fibrous cotton (Figure 10f). The device demonstrates a straightforward and robust method for synthesizing a large-area PPy–modified cotton (PMC) fabric. Moreover, the textiles possess commendable attributes such as sewability and editability, enabling the customizable incorporation of these functional PMC building blocks into a 3D system to achieve highly effective out-of-plane solar vaporization. The 3D design can effectively integrate photothermal textiles into various surroundings, enabling efficient purified water extraction, even from a sandy substrate.

**Figure 11:**
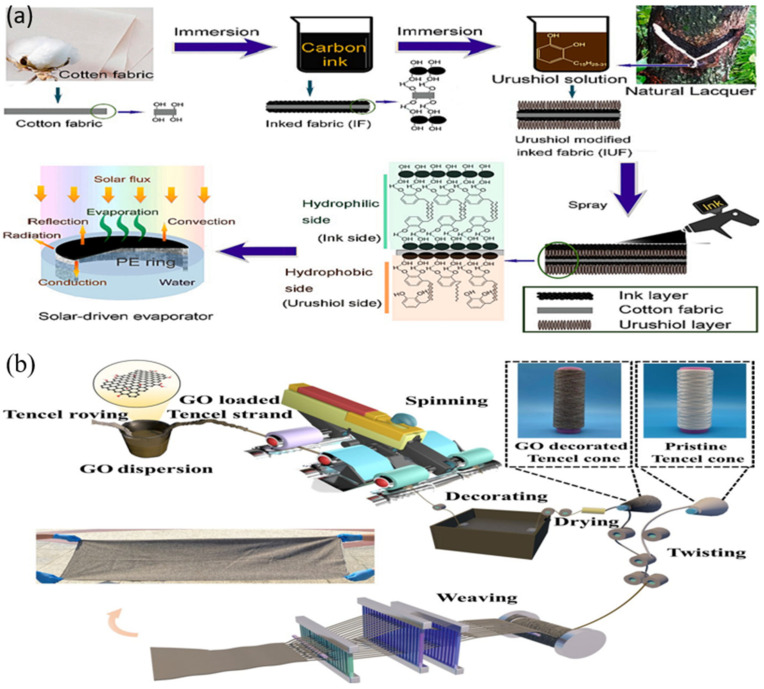
(**a**) Fabrication of the Janus ink/urushiol fabric’s energy balance for the SSG device. Reproduced from ref. [89], copyright 2023, Elsevier. (**b**) The fabrication process of GOT fabric. Reproduced from ref. [90], copyright 2023, Springer Nature B.V.

The fabrication of a Janus ink/urushiol-modified cotton fabric was effectively achieved by Bai W. et al. [89] (Figure 11a) resulting in the development of a fabric with distinct hydrophilic and hydrophobic properties. The Janus fabric exhibited exceptional characteristics in terms of water transportation, mechanical strength, thermal conductivity, and photothermal conversion efficiency. When subjected to simulated solar radiation, the temperature of the Janus cloth rose to 85 °C for 2 min. Furthermore, the apparatus exhibited a notable conversion performance of 94.3%, alongside a remarkable evaporation rate of 1.64 kg m^−2^ h^−1^, with an adequate resistance to UV light and pH levels and a high capacity for rejecting salt in concentrated salt solutions. The system has shown significant efficacy in the purification of wastewater that contains heavy metals and organic dyes. A fabric that possesses many functions, including integrated ISSG and personal thermal control, and offers a sustainable solution for addressing challenges related to water shortages and cold stress was designed by Du H. et al. [90] by a combination of a graphene oxide photothermal absorber and low-cost hydrophilic Tencel fiber as the water supplier (Figure 11b). The regulation of the photothermal conversion, water supply, and moisture diffusion capabilities can be achieved through the modification of the loading, spinning, and twisting parameters. The fabrics that have been combined with whole-yarn-section photothermal capability demonstrate a noteworthy average evaporation efficiency of 90.4% and an impressive evaporation rate of 1.33. The exceptional cost-effectiveness of 222 g h^−1^ USD^−1^ and the durability of 20 washing cycles and 15 operation cycles contribute to the great practicality of this device. In general, the GOT fabric offers a tailored solution for individuals engaging in outdoor activities, requiring garments that offer breathability and insulation, as well as portable devices for accessing clean water.

**Figure 12:**
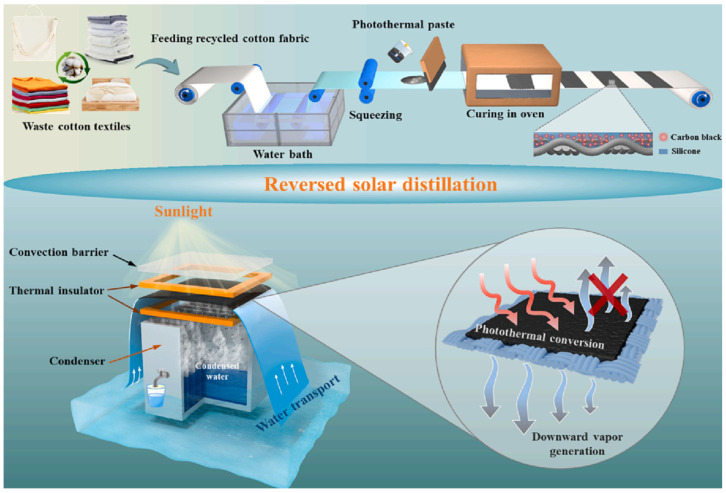
The process of the Janus fabric evaporator with coating process and the procedure of the Janus fabric-based ISSG. Reproduced from ref. [91], copyright 2023, Elsevier.

A novel approach to boost freshwater production by integrating a Janus fabric with thorough thermal control in a reversed ISSG was developed by Gao C. et al. [91]. An inexpensive Janus fabric, which incorporates recycled cotton fabric, was manufactured by applying a layer of carbon black@silicone on the top side using a straightforward and flexible pre-wet coating technique (Figure 12). The Janus fabric possesses effective sealing capabilities that contribute significantly to the prevention of upward vapor formation, hence minimizing optical loss. Consequently, these characteristics of the Janus fabric contribute to the enhanced collection of freshwater in the distillation process. The Janus fabric-based interfacial solar distiller demonstrates a high level of sunlight absorbance, measured at 98.0%, and remarkable performance, with a water production of 1.17 kg m^−2^ h^−1^ and an efficiency of 78% when exposed to one sun (1 kW m^−2^) illumination. The study introduces an innovative design for a high-performance interfacial solar distillation system, which has the capability to be implemented on a large scale and at an affordable rate for industrial production purposes.

**Figure 13:**
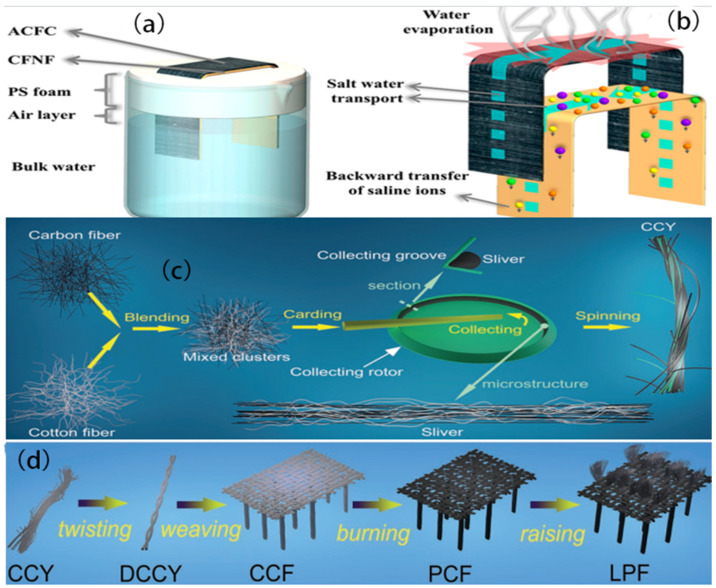
(**a**) The design of the SSG system. (**b**) The transportation routes of water and salt on ACFC and CFNF in ISSG. Reproduced from ref. [71], copyright 2019, American Chemical Society. (**c**) The blending and spinning process of carbon–cotton yarn (CCY). (**d**) The production process of the LPF using the CCY. Reproduced from ref. [73], copyright 2022, Wiley.

In a previous study conducted by Fang Q. et al. [71], a novel ISSG device exhibiting exceptional light-thermal properties was designed by using an activated carbon fiber fabric (ACFC) with tiny architectural structures (Figure 13a,b). However, under optimal conditions, using cotton fiber nonwoven fabrics (CFNFs) in a water supply system can significantly increase the evaporation rate, reaching 1.59 kg m^−2^ h^−1^. Additionally, this system exhibits an impressive conversion performance of 93.3% when exposed to 1 sun irradiation. The significance of achieving optimal alignment between the water supply and vapor evaporation is underscored when maximizing heat utilization and enhancing the efficiency of solar desalination processes. Furthermore, the additional water supply pathway facilitated by the CFNF effectively mitigates salt fouling by the regular removal of salt deposits, ensuring a long-lasting performance of the self-cleaning solar steam generating system. Therefore, the ACFC + CFNF arrangement demonstrates significant potential for utilization in permanent and extremely effective solar desalination.

The continual problem lies in achieving a balance between the expense and freshwater output. To address these issues, separate waste carbon fibers were organized into a yarn that can be stretched and compressed by Wang J. et al. [73]. This yarn was then processed for use in a fabric evaporator, which enables the creation of solar steam. This is achieved through the utilization of two primary textile technologies: blended yarn spinning and multibeam weaving (Figure 13c,d). To enhance the evaporation efficiency of the photothermal device, a bionic fabric structure inspired by the aquatic plant Pistia was devised with the aim of minimizing heat dissipation and maximizing the use of the material. The capacity of the system was enhanced by leveraging the porous structure and gradient capillary effect of the evaporator. By positioning the photothermal component 3.2 cm above the water surface, the evaporation efficiency was significantly increased from 52.70% in its initial state to 88.70%, with an evaporation rate of 1.50 kg m^−2^ h^−1^ under the influence of 1 sun irradiation (1 kW m^−2^). In conjunction with the affordable nature of waste carbon fiber and well-established textile technologies, the overall price of raw materials for the device was decreased to USD 3.54 m^2^. This research presents potential opportunities for an economically viable and scalable method of generating steam from solar power.

### 2.2. Nonwoven Fabrics

Nonwoven fabrics are produced by aligning or randomly arranging staple fibers or filaments to create a fibrous network, which is subsequently strengthened through mechanical, thermal, or chemical processes. They have emerged as a promising and economically viable alternative for ISSG. This can be attributed to their numerous benefits, including the availability of copious raw materials, a straightforward manufacturing method, and the potential for excellent efficiency [54].

**Figure 14:**
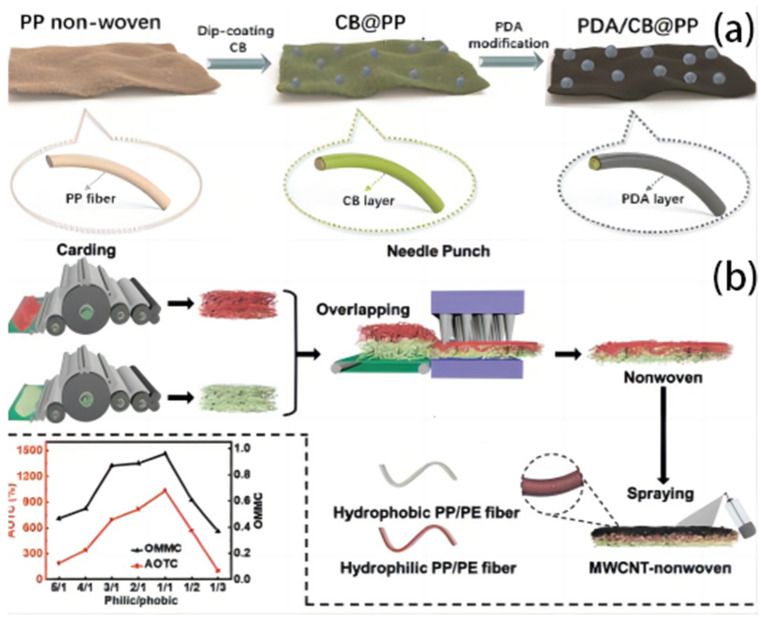
(**a**) Diagram of fabrication of the PDA/CB@PP samples. Reproduced from ref. [30], copyright 2021, Elsevier. (**b**) The fabrication process of MWCNT nonwoven fabric with unidirectional water transfer properties with hydrophilic/hydrophobic fibers. Reproduced from ref. [37], copyright 2021, Wiley.

Sun S. et al. [30] fabricated a carbon black (CB)–polydopamine composite nonwoven fabric (Figure 14a)**.** This method aimed at a cost-effective and scalable PDA/CB@PP composite. In situ PDA polymerization and CB dip-coating were used to make the fabric. A hierarchical framework on the fiber’s outermost layer and CB and PDA synergy produced high light absorbance (>95%), super-hydrophilicity, and energy conversion efficiency. The experimental one-way fluidic PDA/CB@PP photothermally based solar steam evaporator had a 1.68 kg m^−2^ h^−1^ evaporation rate and 91.5% solar steam efficiency. PDA/CB@PP cloth purifies water well, despite the presence of salt. The hydrophilic porous fabric retains water channels and ensures a steady water supply. Additionally, the PDA/CB@PP fabric effectively treats heavy metal- and chemical dye-polluted wastewater.

A solar evaporator that remains in a perpetual state of flotation was constructed by Zhu Y. et al. [37] through the application of multiwall carbon nanotubes onto a bicomponent nonwoven material consisting of polypropylene/polyethylene core–sheath fibers (Figure 14b)**.** The all-fiber structure exhibits a high degree of porosity and ultra-lightness, accompanied by a substantial specific area. The design incorporates features such as enhanced water evaporation efficiency and interconnected passages to facilitate the effective escape of vapor. Additionally, the material should possess a low thermal conductivity in order to minimize heat dissipation. The water transport characteristics of the nonwoven material facilitate its ability to generate a pumping action autonomously. The relationship between the water supply and loss has the potential to expedite the rate of water evaporation of 1.44 kg m^−2^ h^−1^ with an evaporation efficiency of 89.7% under 1 sun irradiation. The fabrication process involves the utilization of inexpensive resources and the implementation of industrialized techniques, resulting in the production of the final product.

The phenomenon of changeable textures and porous clothing being susceptible to pollution is widely acknowledged. However, it is commonly observed that such clothing may be effectively cleansed to eliminate any contaminants, while maintaining their original color and shape without any discernible alterations. Zhu B. et al. [79] designed a washable, stretchable carbon-nanotube-embedded polyacrylonitrile nonwoven fabric using electrospinning technology. The wet fabric has a photo absorption efficiency of 90.8% and high photo-absorption in the 350–2500 nm region. The cloth, coated with a polystyrene foam, showed a high seawater evaporation rate of 1.44 kg m^−2^ h^−1^ under simulated sunlight (1.0 kW m^−2^). Simulations of high saltwater concentrations revealed solid salt deposition on fabric surfaces, leading to a significant decline in the evaporation rate. The washing technique has a minimal impact on the fabric’s shape, photo-absorption, and evaporation, indicating longevity. The use of washable fabrics and parallel PS foams allows for the construction of large-scale outdoor evaporation devices, enabling successful desalination of seawater under natural sunlight.

A nonwoven photothermal cloth with exceptional stability, flexibility, and washability was fabricated by Jin Y. et al. [41] via the process of electrospinning technology. The aim was to enhance the efficiency and longevity of solar steam evaporation. The fabric comprises polymeric nanofibers serving as the matrix, with inorganic carbon black nanoparticles embedded inside the matrix to function as the light-absorbing component. The photothermal fabric, which was enhanced with an optimum carbon loading, exhibits a highly desirable characteristic of being black under water. This fabric can absorb 94% of the sun spectrum, resulting in an impressive solar energy use efficiency of 83% during the process of pure water evaporation. Due to its unique composition and geometry, the fabric exhibits a characteristic resistance to photothermal component loss. It demonstrates exceptional flexibility and mechanical strength, as well as chemical stability, in a wide range of challenging environments, including strong acids, alkaline substances, organic solvents, and saline water. The textile material can undergo handwashing for over 100 cycles without experiencing performance degradation, with an evaporation rate of 1.24 kg m^−2^ h^−1^ and evaporation efficiency of 83%. This characteristic presents a promising approach for effectively removing fouling agents in real-world applications such as solar steam generation and distillation operations.

### 2.3. Electrospun Membranes

**Figure 15:**
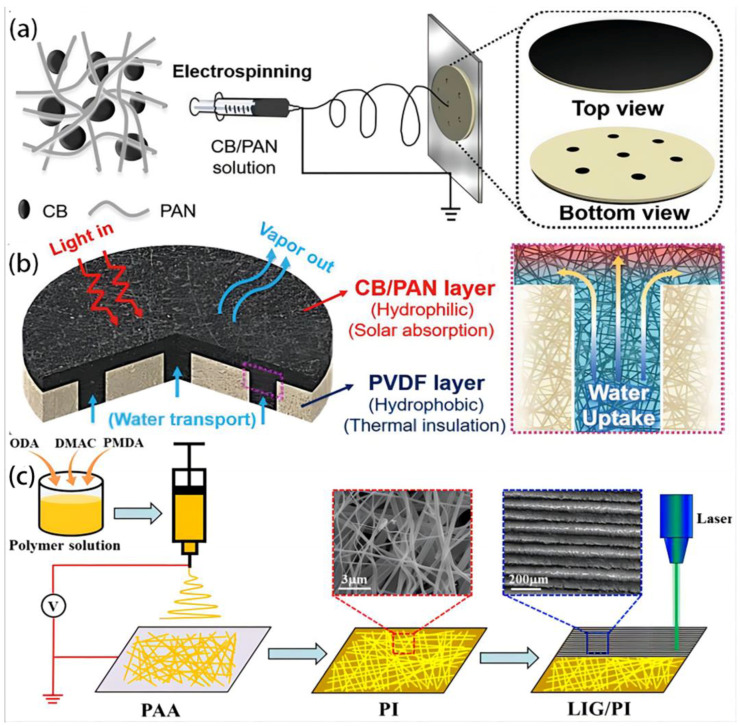
(**a**) The fabrication process and structure of the bilayer SSG device using an electrospinning method. (**b**) The X-section of hydrophilic CB/PAN and hydrophobic holey PVDF layers. Reproduced from ref. [97], copyright 2018, WILEY. (**c**) The design process of PI membrane and LIG/PI membrane. Reproduced from ref. [60], copyright 2020, American Chemical Society.

Electrospun membranes are commonly manufactured using the electrospinning technique [92], which is a highly adaptable textile technique for producing flexible nano-/microfiber membranes [5,93]. It is a process that involves the application of an electric field to create fine fibers from a polymer solution or melt [54,66]. Fiber membranes produced through electrospinning exhibit controlled fiber arrangement, elevated porosity, exceptional mechanical characteristics, and a network of interconnected pores [3]. Moreover, these fibers have diameters that are typically in the range of tens to hundreds of nanometers, resulting in a high surface-area-to-volume ratio and unique properties such as customizable fiber dimensions and pore architectures [55,94], making them promising materials for a wide range of applications, such as being used as a filtration medium with elevated permeability and being used for water treatment [95]. Recently, numerous solar vapor generators utilizing electrospun nanofibers and nonwoven fabrics have been created within the domain of solar vapor generation [5,96]. A cost-effective, buoyant, long-lasting, and expandable evaporator has been developed, incorporating an exposed nanofiber-based dual-purpose framework to generate solar steam efficiently. It is designed by Gao T. et al. [97]. The combined bilayer evaporator is constructed sequentially, starting from the bottom with layers of electrospun hydrophobic polyvinylidene fluoride (PVDF) nanofibers, followed by layers of hydrophilic carbon black/polyacrylonitrile (CB/PAN) composite nanofibers (Figure 15a,b). The presence of a porous hydrophobic PVDF nanofiber layer can be attributed to its inherent poor thermal conductivity. This layer is a floating support and thermal barrier, mitigating irreparable heat loss. The upper layer of the composite nanofiber, consisting of hydrophilic CB/PAN, has a significant solar adsorption capacity throughout a wide range of wavelengths (250–2500 nm), reaching an impressive efficiency of 98.6%. This characteristic enables efficient conversion of solar irradiation into usable heat energy. The CP/P evaporator, when completed, demonstrates a noteworthy solar energy conversion efficiency of 82.0% and evaporation rate of 1.2 kg m^−2^ h^−1^ when subjected to 1 sun illumination. The polymer–nanofiber-based evaporator, which exhibits cost-efficiency, remarkable adaptability, resilience, and capacity, shows significant potential for real-world use in water desalination and disinfection.

Using electrospinning and laser treatment processes, Chen Z. et al. [60] fabricated a photothermal membrane with a three-dimensional (3D) structure. This membrane is composed of laser-induced graphene (LIG) and polyimide (PI) and exhibits significant porosity (Figure 15c). The 3D configuration of the LIG/PI membrane enhances the surface area that is available for evaporation and mitigates the energy dissipation resulting from the scattering of light. The LIG/PI membrane demonstrates a notable evaporation rate of approximately 1.42 kg m^−2^ h^−1^ and a significant solar thermal conversion efficiency of approximately 92.55%. It exhibits long-term evaporation stability in a high-concentration saline solution under one sun illumination, thanks to incorporating an insulating layer and water flow pathways. This durable photothermal membrane has the potential to offer valuable insights for the development of interfacial evaporation systems that are both quick and efficient.

**Figure 16:**
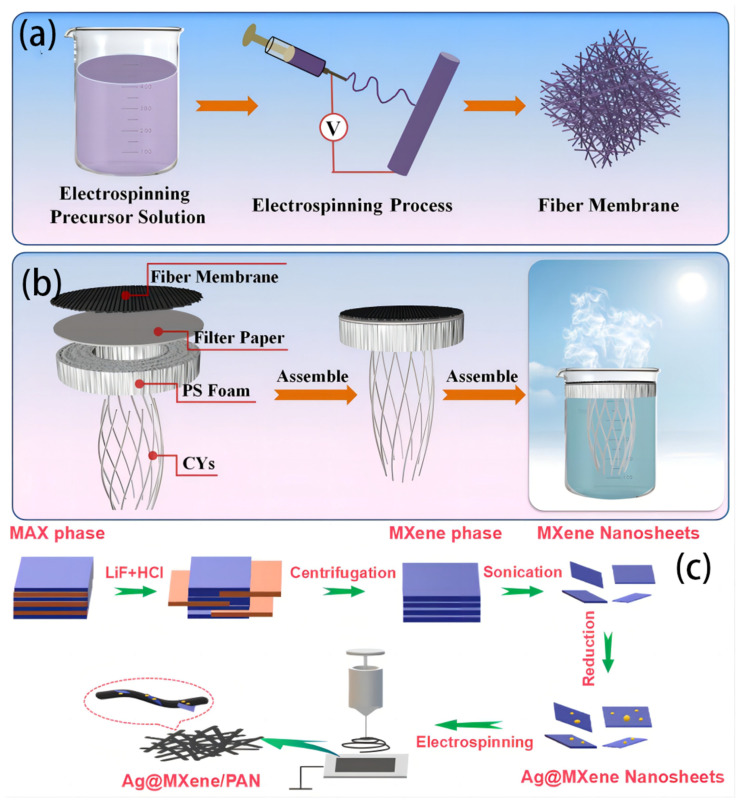
(**a**) The fabrication of (**a**) electrospinning fiber membranes and assembly of (**b**) interfacial water evaporator. Reproduced from ref. [99], copyright 2020, Elsevier. (**c**) The diagram of the design of Ag@MXene/PAN nanofiber membrane. Reproduced from ref. [98], copyright 2021, Elsevier.

The utilization of a solution–electrospun nanofiber membrane has been recognized as a highly suitable framework for solar steam generation. This is attributed to its various advantageous characteristics, including its substantial porosity, which facilitates efficient absorption of light, its significant permeability that enables rapid water transport, and its cost-effectiveness, portable nature, and exceptional adaptability, rendering it extremely useful for real-world applications [98]. An effective and flexible fiber membrane of silicon dioxide/carboxylated multiwalled carbon nanotube/polyacrylonitrile (SiO_2_/MWCNTs-COOH/PAN) was fabricated by Qu Q. et al. [99] by using electrospinning (Figure 16a,b). Subsequently, an interfacial water evaporator was constructed by affixing it onto a filter paper substrate, employing insulating polystyrene (PS) foam as the supporting substance and utilizing cotton yarns for water conveyance. The design offers multiple benefits, including preventing heat dissipation to the bulk water and assuring high evaporation effectiveness. The composite fiber membrane demonstrated a high evaporation rate of 1.28 kg m^−2^ h^−1^ and an impressive photothermal conversion efficiency of 82.52% when exposed to 1 solar irradiation (1000 W m^−2^). In addition, it is worth noting that the composite fiber membrane that was created exhibited remarkable stability in its evaporation rate and efficiency, even after undergoing 20 consecutive evaporation cycles. Therefore, the interfacial water evaporator developed in this study shows exceptional photothermal conversion efficiency, making it a highly viable option for solar-powered saltwater desalination. Additionally, Ag nanoparticle-adorned MXene nanosheets/polyacrylonitrile (Ag@MXene/PAN) nanofiber-based evaporators with a high-efficiency, multipurpose, and unidirectional SSG device was fabricated by Liu H. et al. [98] (Figure 16c). The combination of Ag nanoparticles and MXene nanosheets exhibits several advantageous properties, including enhanced broadband light absorption and heat generation and improved catalytic and antibacterial capabilities. In addition, the membrane possesses the beneficial properties of elasticity and foldability, which allow for creating a three-dimensional evaporator with an enhanced evaporation surface area and optimal light absorption. The developed evaporator exhibits steady evaporation rates throughout a broad range of incident angles, from 30° to 150°. Under 1 sunlight, the maximum evaporation rate approaches 2.08 kg m^−2^ h^−1^, which is significantly higher than the state-of-the-art solution–electrospun nanofiber-based evaporators. The combination of structural adaptability, outstanding efficiency, and multifunctionality renders the developed nanofiber-based evaporator extremely desirable for producing clean water in practical settings.

Plasmonic silver nanoparticles (Ag NPs) used as photothermal coatings and electrospun polyacrylonitrile (PAN) nanofiber membranes used as substrates were utilized by lin Y. et al. [100]. The diameters of the Ag NPs and the light-absorption capabilities of the associated nanofiber membrane were controlled by altering the volume ratios of the glucose and silver ammonia solution. Consequently, the Ag@PAN nanofiber membrane exhibited a notable light-absorption efficiency of 92.8% within the wavelength range of 280–2500 nm. Under irradiations of 1 sun, the evaporation rate and evaporation efficiency were 1.34 kg m^−2^ h^−1^ and 76.0, respectively. The plasmonic nanofiber membrane demonstrated sustained operational stability, as seen by its consistent solar vapor generation capability during a series of 10 cycle tests, without any observable degradation. This research established a foundation for the conceptualization and creation of plasmonic nanofiber membranes with the potential to function as efficient interfacial solar vapor generators. In recent study, an aerogel with a hierarchical pore structure, created using an environmentally friendly bonding and freeze-drying process, was developed by Liu Y. et al. [96] by using electrospinning technique. These aerogels were composed of electrospun nanofibers made from a blend of polyacrylonitrile and carbon nanotubes (PAN/CNTs), and they were developed by combining the synergistic photothermal effect of PDA and CNTs, which significantly increased the light absorption efficiency, reaching 94.8%. The device demonstrated a rapid evaporation rate of 2.13 kg m^−2^ h^−1^ and a high solar–vapor conversion efficiency of 94.5% under one sun irradiation, surpassing the majority of previously published solar vapor generators based on electrospun nanofibers. The aerogel described herein offers a flexible, environmentally sustainable, and economically viable solution for producing clean water.

Xu W. et al.’s [101] study showcases the efficacy of a foldable Janus absorber, manufactured by a sequential electrospinning process, in facilitating reliable and effective solar desalination. The distinctive architecture of the Janus absorber is leveraged to separate the processes of steam generation, namely, solar absorption and water pumping, into distinct layers. The topmost layer consists of a hydrophobic coating of carbon black nanoparticles (CB) embedded in polymethylmethacrylate (PMMA), facilitating light absorption. Meanwhile, the lower layer comprises a hydrophilic polyacrylonitrile (PAN) that is responsible for the water-pumping mechanism. Hence, salt deposition exclusively occurs inside the hydrophilic PAN layer, facilitated by the continuous influx of water, leading to its rapid dissolution. The Janus absorber exhibits a notable level of performance, reaching 72%, which surpasses the performance of many prior absorbers. Additionally, it shows a consistent water output of 1.3 kg m^−2^ h^−1^ over 16 days under 1-sun conditions, a feat that most previous absorbers have not accomplished. The flexible Janus absorber exhibits a distinctive framework that is attained by using an adjustable technique. This absorber is an effective, reliable, and easily transportable solar steam generator, specifically designed for direct sun desalination.

The demonstration of a membrane composed of a hybrid nanofibrous hydrogel-reduced graphene oxide (NHrG) was studied by Zhang L. et al. [102] by using electrospinning technology. The results provide evidence for the existence of intermediate water within the permeable membrane through the observation of the vaporization enthalpy in relation to the saturation of the membrane. Moreover, this finding highlights the significant influence of intermediate water vaporization in reducing the overall vaporization enthalpy. The decreased enthalpy value, in conjunction with many other aspects, such as the enhanced light absorption efficiency facilitated by reduced graphene oxide (rGO) and the formation of a porous hydrophilic network produced by electrospun hydrogel nanofibers, contributed to the development of a very effective solar-driven interfacial evaporator. The NHrG membrane exhibited a maximum evaporation rate of 1.85 kg m^−2^ h^−1^, accompanied by a notable energy conversion efficiency of 95.4% when subjected to 1 sun irradiation. Furthermore, the evaporator demonstrated exceptional desalination efficacy when employed for the treatment of unpolluted seawater, effectively eliminating salt and heavy metal ions.

### 2.4. Knitted Fabrics

**Figure 17:**
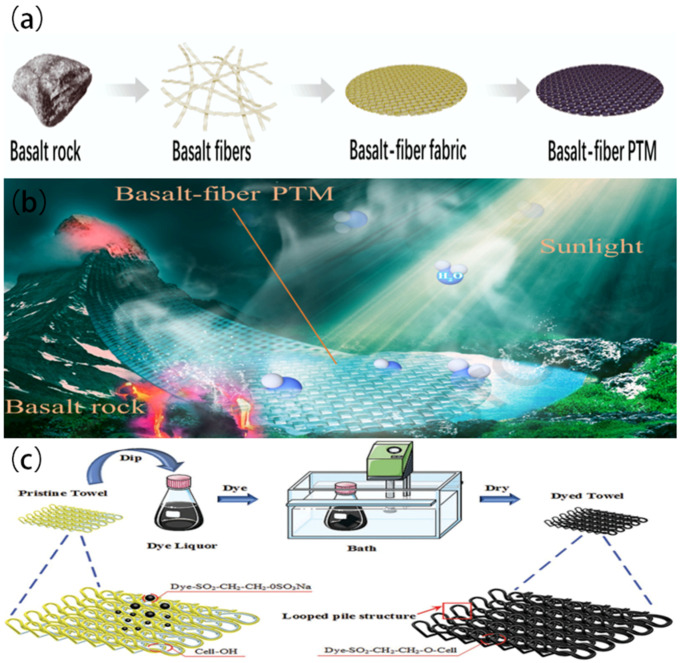
(**a**) A diagram depicting the procedure for the fabrication of the basalt fiber (PTM). (**b**) The design of the basalt fiber PTM in photothermal evaporation system. Reproduced from ref. [103], copyright 2021, Elsevier. (**c**) Diagram of the dyeing steps. Reproduced from ref. [8], copyright 2019, WILEY.

Knitting is a textile production method that involves creating interlocking loops of yarn or thread using knitting needles or machines. Knitted fabrics have distinct characteristics and are widely used in various applications. A cost-effective and adaptable photothermal membrane (PTM) was fabricated by Wan P. et al. [103] from basalt fibers derived from natural basalt by using knitting technology (Figure 17a,b). The synthesis of the basalt fiber PTM involves the processes of fiber formation and heating. Due to its unique chemical composition and structural characteristics, the PTM exhibits exceptional durability and corrosion resistance in challenging conditions, including harsh acids, organic solvents, and alkalis. The investigation focused on the techniques of water evaporation, with particular attention given to the floating-with-insulator model. This technique shows the least heat dissipation and the fastest rate of water evaporation. The optimization of the basalt fiber fabric involves the processes of carbonization and vaporization. Through these processes, a vaporization rate of 1.50 kg m^−2^ h^−1^ and an energy utilization efficiency of 82.5% were attained for the evaporation of clean water. The PTM made from basalt fiber exhibits a notable resistance to efficiency degradation, allowing it to withstand hand washing for a minimum of 30 cycles. The manufacturing method’s scalability enables it to cater to commercial demands effectively.

An easily manufactured, cost-effective, and highly reliable solar-driven evaporator was fabricated by Yang Y. et al. [8] by utilizing a dyed cotton towel with a hollow conical shape. After the dyeing process (Figure 17c), the reactive dye molecules underwent diffusion into the cotton fabric and established robust covalent bonds with the fibers. This resulted in the secure attachment of light-absorbing components onto the substrate. Utilizing a looping pile architecture in towels and an ordered arrangement in yarns facilitates the expansion of the evaporator’s surface area. The cotton towel’s hollow cylindrical structure demonstrates a notable capacity to mitigate heat dissipation to the surrounding environment while maintaining its ability to absorb light. The 3D vapor generator demonstrates an evaporation rate of 1.40 and 1.27 kg m^−2^ h^−1^ for pure and saline water. Moreover, it offers a dependable approach for processing real-world water sources, including saltwater and dyeing wastewater. Hence, a cost-effective water evaporation system powered by solar energy presents a supplementary method for achieving optimal vapor generation and water purification in real-world scenarios.

Wang F. et al. [47] developed an innovative solar steam generation system utilizing a hollow spacer fabric (HSF) that was modified by incorporating chitosan and applying a reduced graphene oxide coating onto its outermost layer. The improved BHSF exhibits enhanced efficiency in terms of water transport and mechanical properties due to its porous textile fabric structure, which provides superior thermal insulation. It demonstrates an elastic modulus of up to 733 kPa at a strain of 70% and a low thermal conductivity of 0.08 W m^−1^ K^−1^. When subjected to sun irradiation with a power density of 1 kW/m^−2^, this solar generator exhibits an evaporation efficiency of 86%, with an evaporation rate of 1.44 kg m^−2^ h^−1^. The improved BHSF exhibits exceptional resistance to salt, particularly in high-saline solutions, due to its distinctive cylindrical structure and the presence of a well-aligned array of large channels measuring 2 mm in size. This distinctive design provides extra pathways with minimal tortuosity, facilitating efficient evaporation. The BHSF exhibits promising prospects for the large-scale production of a cost-effective solar still. This solar still possesses exceptional mechanical versatility, physicochemical durability, and a high solar energy conversion efficiency. These attributes make it suitable for various uses, such as power generation, desalination, and steam sterilization.

Pursuing an ecologically sustainable method for acquiring cellulose nanofiber and fabricating an evaporator based on aerogel represents a practical necessity. He M. et al. [104] fabricated a novel cellulose hybrid aerogel ISSG that is both stable and green from waste cotton fibers. The aerogel was composed of polyethyleneimine cross-linked carbon nanotubes and cellulose nanofibers (PEI@CNTs/CNFs), and it shows a notable solar steam generation rate of 1.9 kg m^−2^ h^−1^, with a light vapor conversion rate of 91.4% when exposed to 1 sun irradiation. Furthermore, the evaporator can ensure consistent and enduring efficiency for solar steam generation even after undergoing extensive long-term testing. In addition, the device can treat many types of wastewater, such as dye sewage, toxic ion wastewater, and seawater. The above characteristics of the cellulose-based composite aerogel provide a highly effective method for solar-powered water filtration, regeneration, and desalination. Regenerative cellulose aerogels (CAs), functionalized with polydopamine (PDA), were developed by Liu H. et al. [28] by using an environmentally friendly and practical approach to facilitate the production of clean water. By initiating the polymerization of PDA on the surface, a material known for its remarkable photothermal conversion capabilities and water-purifying properties, the result demonstrates a notable light absorption efficiency of 96.5% and an evaporation rate of 2.74 kg m^−2^ h^−1^ under 1 sun irradiation. In the context of solar steam production, it has been observed that a solar steam generator with an increasing height can absorb energy from the surrounding atmosphere, hence enhancing the process of vapor creation. Renewable cellulose-based aerogels have inherent characteristics that facilitate effective water evaporation. These attributes, together with their cost-effectiveness and recyclability, hold significant potential in mitigating both energy consumption and the environmental impact associated with cotton fabric.

## 3. Salt Rejection and Accumulation in Textile Material-Based ISSG Systems

The continuous process of saltwater evaporation results in the deposition of salt on the surface of photothermal conversion materials. This salt buildup has the potential to obstruct the steam channels and subsequently diminish the rate of water evaporation. The avoidance of salt accumulation on solar-driven evaporators is a crucial consideration in their practical implementation [105]. However, salt accumulation is frequently seen in sun desalination operations, particularly in brine with high salt levels. The presence of precipitated salt has the potential to obstruct water pathways, impede the passage of vapor through escape channels, diminish the capacity of the evaporation surface to absorb light, and, thus, lead to decreased effectiveness in evaporation while operating continuously in saline water [38]. However, the accumulation of salt on the absorbers during the process of desalination is a significant challenge, as it has the potential to degrade and disrupt the ongoing production of steam. Several effective solutions have been described in the literature to mitigate salt buildup. These methods can be categorized into two main types: hydrophilic and hydrophobic designs, as well as contactless architectures. The hydrophilic design incorporates an extra water supply channel to facilitate the improvement in fluid convection. This convection process effectively dilutes the concentrated brine that is generated within the absorbers [46,75]. However, for better performance and higher efficiency of ISSG devices in the desalination process, the following key factors should be considered during the design and the operation of the system as well: (i) The optimal selection and modification of the photothermal materials with high solar absorptivity and low thermal emittance to optimize heat absorption and decrease heat loss; (ii) Enhancing the efficiency of the heat transfer surfaces by optimizing their design, integrating heat pipes, or using improved heat transfer fluids; (iii) Integrating the SSG device with other desalination methods to increase the overall efficiency. Coupling SSG with processes like multi-effect distillation, membrane distillation, or thermal vapor compression may boost energy usage and water recovery rates; (iv) Consistent cleaning and upkeep of the photothermal material surface and vigilant monitoring of system components may effectively avoid deterioration, fouling, or scaling. Enduring efficiency requires the presence of long-term stability and dependability.

Recently, numerous meticulously planned buildings have been documented, with the aim of mitigating the issue associated with surface salt precipitation; these constructions achieve this by using salt-rejecting photothermal devices that facilitate the dispersion of collected salts back to the source water [106]. The removal of solid salts from membranes can be achieved through washing. However, it should be noted that certain photothermal membranes, including fabrics, cellulose membrane, and aerogel, are susceptible to damage when subjected to the washing process [38]. This is primarily attributed to the exfoliation of nanoparticles and the lack of sufficiently big micronized channels. To enhance the efficiency of washing and regeneration processes, it is imperative to focus on the development of innovative photothermal membranes that possess substantial micronized channels and are resistant to potential exfoliation of the photothermal nanomaterials [79].

Table 1 presents a summary of the outcomes and outstanding features of textile material-based ISSG devices, including their evaporation rates and evaporation efficiency when subjected to 1 solar irradiation. However, due to the unique properties of the textile materials, such as thinness, a lightweight nature, flexibility, comfort, porous nature, water transfer and evaporation, a large surface area, and permeability, the listed ISSG textile-based devices generally showed an outstanding evaporation efficiency ranging from 80 to 98%, which is very competitive compared to that of many other ISSG systems. The evaporation rate was in the range of 1.18–2.32 kg m^−2^ h^−1^, which is in the acceptable range for the high-performance ISSG devices reported in the literature. Among the studied systems, the vertical polypyrrole nanowire-coated fabric ISSG device demonstrated a significantly higher evaporation efficiency of 98.56% with an unparalleled evaporation rate of 2.32 kg m^−2^ h^−1^, which is the highest compared with the other devices reported in the literature. On the other hand, the MWCNTs-COOH/cotton fabric showed a relatively high evaporation efficiency of 86.0 1% with the lowest evaporation rate of 1.18 kg m^−2^ h^−1^.

## 4. Drawbacks and the Challenges of Textile Material-Based ISSG Devices

The efficiency of solar-driven evaporation systems can be influenced by factors such as weather conditions, temperature, and the availability of sunlight. Additionally, the scalability and productivity of solar-driven evaporation systems need to be carefully considered to meet the demands of large-scale desalination projects. The following criteria are the most significant in terms of impeding their potential for future industrial applications [75]:

1. Scalability: Many of the current solar interfacial evaporation techniques are still at the laboratory or small-scale prototype stage. Scaling up the technology to an industrial level presents challenges in terms of maintaining efficiency, cost-effectiveness, and system stability. Further research and development are needed to optimize the design and manufacturing processes for large-scale implementation.
2. Adaptability: Solar interfacial evaporation systems need to be adaptable to various environmental conditions and water sources. Factors such as the solar intensity, temperature variations, water quality, and impurities can affect the performance of the system. Developing adaptable and robust systems that can operate effectively under different conditions is essential.
3. Stability and durability: Solar interfacial evaporation systems operate in harsh environments, including exposure to sunlight, saltwater, and other contaminants. The materials used in these systems should be stable, durable, and resistant to degradation over extended periods. Ensuring long-term stability is crucial for the practical and reliable operation of this technology.
4. Integration with existing infrastructure: To facilitate widespread adoption, solar interfacial evaporation systems need to be compatible with existing water treatment and distribution infrastructure. Integration with existing systems, such as storage and distribution networks, would enable the seamless implementation and utilization of the produced freshwater.

Addressing these limitations requires ongoing research and development efforts through interdisciplinary collaboration, as well as technological advancements. With continued progress, it is possible to overcome these challenges and unlock the full potential of solar interfacial evaporation for future industrial applications in freshwater production.

## 5. Future Prospects and Recommendations

The utilization of textile materials in solar steam generation exhibits favorable future possibilities. Textile substances possess distinct benefits, including adaptability, being lightweight, expansion potential, and affordability, rendering them well-suited for solar steam-generating technologies. Below are some potential uses of textile materials in this domain:

1. Solar steam generation can be employed for desalination, wherein saltwater or brackish water is transformed into potable water via evaporation and condensation. Textile materials, such as woven or knitted materials with appropriate surface coatings, can be highly effective solar absorbers for capturing solar energy and producing steam for desalination. Due to their adaptability and capacity for expansion, they are well-suited for solar desalination processes on a wide scale.
2. Textile materials can be employed in the production of solar steam generation systems. Textiles featuring sophisticated coatings or fibers that are modified for specific functions can be integral in constructing solar collectors, steam chambers, or heat transfer systems. Integrating textile manufacturing knowledge can improve the efficiency and performance of solar steam generation systems.
3. Further gains can be achieved through ongoing research and innovation in textile materials and their utilization in solar steam generation. This encompasses the creation of innovative textile architectures, surface treatments, or substances with heightened sun absorption capabilities, enhanced heat conduction attributes, and augmented longevity. Textile-based solar steam-generating methods can be further enhanced by advancements in nanotechnology, intelligent fabrics, and improved manufacturing techniques.
4. It is crucial to acknowledge that textile materials present promising opportunities for generating solar steam. However, there remain obstacles to address, including the need to enhance textile-based systems’ efficiency, longevity, and adaptability. Additionally, ensuring stability over time in harsh environments and effectively integrating these systems into current structures pose significant challenges. Nevertheless, with continuous research and improvement endeavors, the utilization of textile materials in generating steam from solar energy is expected to broaden. This will aid in providing sustainable energy solutions and tackling worldwide issues related to water shortages, energy accessibility, and environmental sustainability.

## 6. Conclusions

Solar interfacial evaporation has emerged as a technology that is receiving considerable interest due to its potential to effectively mitigate the issue of worldwide freshwater scarcity. There are many attractive characteristics that are available in textile materials. They are very appropriate for a wide range of products and uses because of their thinness, lightweight nature, flexibility, comfort, porous nature, water transfer and evaporation, large surface area, and permeability. However, recently, the use of textile materials in ISSG showed encouraging results in a variety of applications for wastewater purification and desalination. This comprehensive review examines several ISSG textile-based device materials, and the results demonstrate exceptional evaporation efficiencies, ranging from 80% to 98%, with an evaporation rate in the range of 1.18–2.32 kg m^−2^ h^−1^ for ISSG devices with outstanding performances. However, this level of performance is very competitive when compared to other ISSG systems.

## Figures and Tables

**Table 1:** The performance of textile material-based ISSG devices.

Textile Material	Materials	Efficiency (%) Under 1 Sun	Evaporation Rate (kg m^−2^ h^−1^)	Ref.
Woven fabrics				
	PDA/PPy nanofibers/Flax fabric	87.40	1.37	[45]
	PAN/Cotton yarns	83.70	1.87	[75]
	MXene/PET fabric	80.00	1.22	[74]
	PAN/Spacer fabric	86.00	1.43	[47]
	VPPyNW fabric	98.56	2.32	[81]
	CB/SA/Ramie fabric	96.60	1.81	[17]
	CNTs/Cotton fabrics	95.70	1.59	[34]
	MXene/CNT/Cotton fabric	88.20	1.35	[58]
	CB/Cotton fabric	88.90	1.33	[53]
	Cs_x_WO_3_ ink/Cotton fabric	86.80	1.56	[33]
	Candle soot/Linen fabric	90.00	1.44	[31]
	Cu/Polyester textile	88.00	1.52	[82]
	MU/PAN textile	89.20	1.36	[83]
	Nafion coating/Cotton cloth–NCC	89.90	1.52	[85]
	Polyester/Cotton Janus fabric	86.30	1.37	[84]
	PPy fabric/PSHF	91.68	1.49	[107]
	TiO_2_ nanorods/PAN fabric	93.00	1.42	[42]
	PPy/Cotton fabric	82.99	1.20	[72]
	PAN cloth/PAN fiber cloth	93.70	1.43	[86]
	RGO/Cotton fabric	N/A	1.47	[70]
	Au-CNT/Cotton fabric	N/A	2.19	[24]
	MXene/CNT/Cotton fabric	88.20	1.35	[58]
	MXene/Cotton fabric	83.10	1.38	[69]
	MWCNTs-COOH/Cotton fabric	86.01	1.18	[21]
	PEGylated MoS_2_/Cotton fabric	80.50	1.30	[108]
	PPy/Cotton fabric	N/A	1.54	[88]
	PAN/Cotton composite fabrics	82.00	1.25	[87]
	PANI/Cotton fabrics	89.90	1.94	[109]
	Janus ink/Urushiol cotton fabric	94.30	1.64	[89]
	PPy/Janus cotton fabric	91.00	1.45	[110]
	PAN/Waste carbon fiber	88.70	1.50	[73]
	PAN/Composite Tencel (GOT) fabric	90.40	1.33	[90]
	(Ag NPs)/Cotton fabric	91.00	1.66	[111]
Nonwoven fabrics				
	PDA/CB@PP nonwoven fabric	91.50	1.68	[30]
	CNT/PAN nonwoven fabrics	90.80	1.44	[79]
	PP/PE nonwoven/MWCNTs	89.70	1.44	[37]
	CB/Nylon fabric	83.00	1.24	[41]
	PAN/Cotton fiber nonwoven fabric	93.30	1.59	[71]
	MnCDs@PPy/nonwoven cotton fabric	96.40	1.68	[112]
Electrospun membranes				
	CB/PAN//PVDF composite layer	82.00	1.20	[97]
	G/PPy/LIG/PI membrane	92.55	1.42	[60]
	PAN/CNTs nanofiber	94.50	2.13	[96]
	CNP/PCL nanofiber composites	N/A	1.95	[113]
	CNT/PCL nanofiber composites	N/A	2.00	[113]
	FIP-PZ/MOF fabrics	94.20	1.50	[114]
	Ag@MXene/PAN nanofiber membrane	92.40	2.08	[98]
	Ag@PAN nanofiber membrane	76.00	1.34	[100]
	SiO_2_/MWCNTs-COOH/PAN fiber membrane	82.52	1.28	[99]
	GO/PVA EFMs membrane	94.20	1.42	[5]
	CNFs/PAN/TPA nanofibrous membrane	89.50	1.36	[16]
	CNT/PVDF/PVP nanofiber	86.10	1.37	[38]
	Co_3_S_4_HP/PAN membrane	86.50	1.26	[115]
	CNTs@SiO_2_ Nanofibrous Aerogels	98.00	1.50	[11]
	rGO/PAN membrane	89.40	1.46	[93]
	CB/PAN membrane	72.00	1.30	[101]
	rGO/NHrG membrane	95.40	1.85	[102]
	GO/PVA EFMs nanofiber mats	90.00	1.40	[5]
	Carbonized ultrafine PAN fibers	81.71	1.33	[92]
	Chinese ink/PLA fibers	81.00	1.29	[94]
Knitting fabrics				
	Carbonized basalt fiber fabric	82.50	1.50	[103]
	3D dyed black cotton towel	72.00	1.40	[8]
	Chitosan/GO/3D spacer fabric	86.00	1.44	[47]
	PEI@CNTs/Waste cotton fabric	91.40	1.90	[104]

N/A: data are not available.

## Data Availability

Not applicable.
